# Post-Stroke Rehabilitation: Neurophysiology Processes of Bilateral Movement Training and Interlimb Coupling—A Systematic Review

**DOI:** 10.3390/jcm14113757

**Published:** 2025-05-27

**Authors:** Jan A. Kuipers, Norman Hoffman, Frederick R. Carrick, Monèm Jemni

**Affiliations:** 1 The Carrick Institute, Cape Canaveral, FL 32920, USA; drnorm@hoffmanwellness.com (N.H.); drfrcarrick@post.harvard.edu (F.R.C.); monemj@hotmail.com (M.J.); 2 Centre for Mental Health Research, University of Cambridge, Cambridge CB2 1TN, UK; 3 College of Medicine, University of Central Florida, Orlando, FL 32827, USA; 4 Burnett School of Biomedical Science, University of Central Florida, Orlando, FL 32827, USA; 5 MGH Institute for Health Professions, Boston, MA 02129, USA; 6 Faculty of Physical Education, Ningbo University, Ningbo 315000, China

**Keywords:** stroke rehabilitation, post-stroke rehabilitation, bilateral movement training, cross-education, interlimb coupling, interlimb transfer

## Abstract

**Background:** Bilateral movement training (BMT) and interlimb coupling have emerged as promising neurophysiologically-based rehabilitation approaches for stroke survivors. However, the underlying mechanisms and optimal implementation strategies remain incompletely understood. This systematic review explored the neurophysiological principles underlying BMT and interlimb coupling interventions that led to positive clinical post-stroke rehabilitation outcomes, focusing on identifying the most effective bilateral and interlimb movement strategies. **Methods:** A 10-year literature search (2014–2024) following PRISMA guidelines was conducted across PubMed, Cochrane, and Google Scholar databases using keywords including stroke rehabilitation, bilateral movement training, cross-education, interlimb coupling, and interlimb transfer. Studies were included if they involved human subjects, clinical trials, stroke survivors, and described bilateral training protocols. Data extraction focused on neurophysiological mechanisms, intervention characteristics, and clinical outcomes. Quality assessment was performed using validated methodological appraisal tools, including the Newcastle-Ottawa Scale and Cochrane RoB 2.0. **Results:** Of 199 initially identified studies, 28 met inclusion criteria for detailed analysis. BMT demonstrated effectiveness in enhancing motor recovery by engaging neurophysiological mechanisms, including central pattern generators, interhemispheric coupling, and cortical disinhibition. High-intensity BMT provided significant gains for individuals with moderate to severe impairments, while low-intensity training benefited early recovery stages. Interventions incorporating task-specific exercises, robotic assistance, sensory enhancement, and virtual reality showed particular promise for addressing motor recovery complexities. However, significant research gaps were identified, including limited understanding of individualized responses to BMT, insufficient research on combined upper and lower limb training, and minimal integration of advanced technologies. **Conclusions:** BMT and interlimb coupling play critical roles in post-stroke rehabilitation by facilitating neural plasticity and interlimb coordination. Integrating robotic assistance, sensory enhancement, and virtual reality with BMT offers a robust framework for maximizing rehabilitation outcomes. Future research should prioritize longitudinal studies, personalized rehabilitation approaches, technology integration, and stratified interventions tailored to individual needs to optimize neuroplasticity and enhance quality of life for stroke survivors.

## 1. Introduction

Over the past two decades, a substantial body of research has significantly advanced our understanding of bilateral movement training, cross-education, and stroke rehabilitation, placing us at the forefront of this rapidly evolving field. This research is of paramount importance, as it underpins the effectiveness of these approaches in post-stroke motor recovery.

Bilateral movement training is a stroke rehabilitation approach to movements that consists in using both limbs to perform symmetrical and nonsymmetrical movements. This approach is characterized by the coordinated engagement of both limbs simultaneously, promoting functional recovery, particularly following neurological conditions such as stroke. This method leverages neural coupling between the brain hemispheres, promoting interhemispheric communication and synchronization to facilitate motor recovery in the affected limb [1,2]. Studies have shown that bilateral movement training can enhance strength, dexterity, and the functional use of the paretic limb, thereby increasing neural network efficiency [3]. Bilateral movement training maximizes recovery outcomes, often integrated with task-specific training and technology-assisted interventions [4]. Cross-education in stroke rehabilitation refers to improvements in motor performance of the untrained limb when training the contralateral limb.

This concept is grounded in the principles of neural plasticity and interhemispheric transfer. It occurs due to the activation of bilateral motor cortices and transcallosal communication during unilateral exercise [5]. Cross-education is advantageous for patients with significant impairments in one limb, as it utilizes the unaffected limb to facilitate recovery in the affected limb. Mechanisms include increased cortical excitability, enhanced motor unit recruitment, and improved coordination and strength in the untrained limb [6].

Harjpal et al. and Stewart emphasize the growing use and effectiveness of bilateral movement training in post-stroke motor recovery [2,7]. Cauraugh further supports this view, identifying that coupled protocols, such as combining bilateral training with EMG-triggered neuromuscular stimulation, demonstrated particularly significant effects [8]. Liu et al. caution that not all rehabilitation methods, including bilateral arm training, have shown superiority over other methods and that combinations of methods can result in better outcomes [9]. Timmermans et al. [10] emphasize the potential of integrating technology-assisted training with basic and clinical science research in stroke recovery and rehabilitation for improved outcomes.

Evidence suggests that bilateral movement therapy is an effective alternative for training affected limbs mainly after stroke, particularly when minimal or no active movement is possible. It is important to note that most studies on bilateral training have focused on the arms and hands.

Several authors have indicated that interlimb connections can yield positive training effects in stroke rehabilitation [11]. Other papers emphasizing the importance of interlimb coupling in stroke rehabilitation include Zehr et al., who highlighted the significant impact of treatment studies, particularly for stroke [12]. Li et al. proposed a protocol for analyzing the clinical benefits of interlimb-coordinated intervention in gait recovery and rehabilitation [12,13]. Maceira-Elvira et al. and Arya et al. explored advanced technologies in stroke rehabilitation, such as brain–computer interfaces and interlimb coupling protocols [14,15].

Although humans have adopted a hindlimb strategy for locomotion, the central nervous system retains a capacity for quadrupedal movement, utilizing all limbs synchronously. Numerous studies have examined the effectiveness of combined upper and lower limb movement training in stroke rehabilitation. Khan et al. found that combining task-oriented approaches with occupational therapy and physical therapy can be effective, while Keeling et al. highlighted the potential benefits of integrating robotic rehabilitation with other therapeutic approaches [16,17]. Hesse et al. discussed the promise of robot-assisted rehabilitation, with Hesse noting its potential for both upper and lower limb rehabilitation [18]. Cauraugh and Kang and Hatem et al. emphasized the importance of combined interventions, with Cauraugh and Kang specifically noting the benefits of coupled bilateral training [19,20]. French et al. and Yoon et al. provided comprehensive overviews of various rehabilitation strategies, with French focusing on task-oriented repetitive training and Yoon highlighting the effectiveness of constraint-induced movement therapy and mirror therapy [21,22]. However, research exploring the efficacy of interlimb coupling, including quadrupedal or crawling movements, as part of stroke rehabilitation strategy is sparse [15,23].

There is a need for a more precise description of interlimb coupling and bilateral movement training strategies and their underlying neurophysiological concepts. Understanding the underpinning neurophysiology of bilateral movement training is critical because it informs therapeutic protocols by elucidating how interhemispheric neural coupling and cortical reorganization promote motor recovery, thus optimizing stroke rehabilitation interventions [24]. Although several authors have demonstrated that bilateral and interlimb strategies can yield favorable outcomes in stroke rehabilitation, most studies have focused on bilateral movement training for the upper limbs [25]. Few have identified specific movement strategies or protocols that enhance bilateral and interlimb movement strategies [23,25]. For clinicians involved in stroke rehabilitation, it is not always clear which interlimb strategy and protocols are most effective. Bilateral movement training has the most potential to work in conjunction with other therapeutic interventions, based on the current body of research. This knowledge gap presents an exciting challenge and opportunity for further exploration and innovation in the field.

Therefore, the primary objective of this review is to highlight the underlying neurophysiological principles of some of the most promising bilateral and interlimb movement strategies that led to positive clinical post-stroke rehabilitation outcomes.

## 2. Method

### 2.1. Search Strategy and Selection Criteria

A literature search followed PRISMA guidelines (for PRISMA 2020 checklist, see Supplement File S1) to include papers published between 2014 and May 2024, utilizing three databases: PubMed, Cochrane, and Google Scholar. This ten-year time frame was retained as an update of the previous ten-year period. This ten-year period also provides a more accurate reflection of recent advances in bilateral training interventions, capturing the evolving understanding of neurophysiological mechanisms underlying changes in bilateral movement training, particularly in light of new imaging and molecular tools. The concepts of bilateral movement training, cross-education, interlimb coupling, and interlimb transfer began to appear in the literature in the early 2000s, thanks to the groundbreaking work of Stewart et al., Whitall et al., and Cauraugh and Summers [2,26,27].

**Figure 1:**
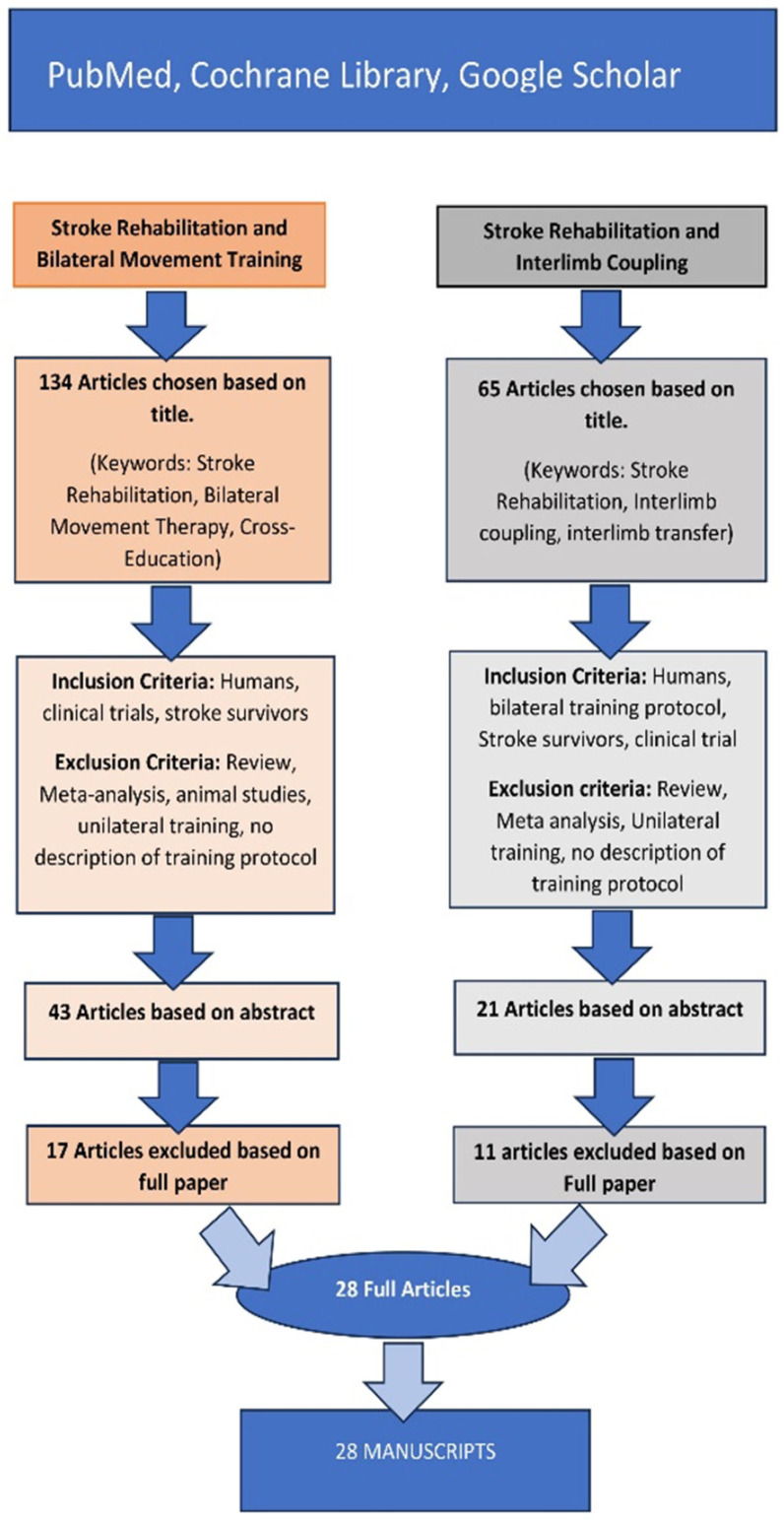
PRISMA flow diagram for research and selection process. Twenty-eight articles were retained and the review considered all of them.

The databases were screened using the keywords stroke rehabilitation, post-stroke rehabilitation, bilateral movement training, cross-education, interlimb coupling, and interlimb transfer. The entire review protocol is shown in Figure 1 (PRISMA flow diagram for the search and selection process). A first screening process yielded 134 articles on stroke rehabilitation, bilateral movement therapy, and cross-education, as well as 65 studies on stroke rehabilitation, interlimb coupling, and interlimb transfer. After removing duplicates, titles and abstracts were independently screened by two reviewers. Disagreements were resolved by discussion or consultation with a third reviewer.

Further inclusion criteria were applied to the 134 studies on stroke rehabilitation, bilateral movement training, and cross-education, specifically focusing on human subjects, clinical trials, and stroke survivors. Exclusion criteria included reviews, meta-analyses, animal studies, unilateral training, and studies with no training method or protocol described. These criteria narrowed the number of papers to 43 based on their abstracts. Twenty-six papers were further excluded after reading the full papers, resulting in the retention of 17 papers related to post-stroke rehabilitation, bilateral movement training, and cross-education.

The inclusion criteria applied to the 65 post-stroke rehabilitation, interlimb coupling, and interlimb transfer studies were as follows: humans, stroke survivors, clinical trials, and bilateral training protocols. Studies were excluded if no exercise protocol was described and unilateral training methods were used. These criteria reduced the number of papers to 21 based on their abstracts. Further exclusion of articles after reading the full papers reduced the total to 11 post-stroke rehabilitation, interlimb coupling, and interlimb transfer-related full papers that were retained.

### 2.2. Standardized Data Extraction

Two reviewers independently extracted data using a structured and standardized data extraction form specifically designed to address the following: exploring the underlying neurophysiological principles of some of the most promising bilateral and interlimb movement strategies that led to positive clinical post-stroke rehabilitation outcomes. The extraction form systematically documented details of each study’s design, sample size, participant clinical and demographic characteristics, neurophysiology underpinning the interventions, interventions administered (including type, intensity, frequency, and duration), and clinical or functional outcomes assessed. Extracted data focused explicitly on the identification and synthesis of the underlying neurophysiological principles of the diverse group of bilateral training methods.

### 2.3. Quality Assessment

The quality assessment of included studies was independently conducted by two reviewers using validated methodological appraisal tools matched to the study designs. A risk of bias assessment was performed for the included studies using the Newcastle–Ottawa Scale, Cochrane RoB 2.0, or modified Cochrane ROBINS I (2016) assessment tools, depending on study type (see Supplement File S2 for results). The results of this assessment are presented in a table as Supplement File S2, and a narrative summary has been added to the Results and Discussion sections. Given the anticipated methodological diversity and heterogeneity among studies, a quantitative meta-analysis was not planned. Additionally, no statistical methods, such as funnel plots, were applied to assess publication bias due to the expected variability in study designs and outcomes. Instead, findings were synthesized qualitatively, highlighting key themes, consistent patterns, and critical gaps identified across the included studies. Given the focus on reviewing underpinning neurophysiological principles, this review systematically considered studies conducted across diverse settings, including controlled clinical trials and real-world rehabilitation environments. To account for variability in intervention type, data extraction included detailed documentation of rehabilitation protocols, specifying whether interventions were administered under strictly controlled conditions or within routine rehabilitation programs. Treatment intensity, frequency, duration, and outcomes were systematically analyzed, as well as any explanation of neurophysiological processes by the different authors, to determine differences in the related neurophysiological underpinnings. This approach provided a comprehensive understanding of the neurophysiology underlying the effects of various bilateral training methods.

## 3. Results and Discussion

### 3.1. Bilateral Movement Training

Bilateral movement training (BMT) has gained prominence as a beneficial rehabilitation technique for stroke patients experiencing upper extremity paresis. This approach involves the simultaneous movement of both arms, demonstrating efficacy in enhancing motor function and facilitating recovery [2,28,29]. The definition of bilateral movement training was popularized by Cauraugh and Summers [27], who discuss the concept in their paper. They delve into the importance of this type of training, which involves coordinated movements of both sides of the body simultaneously or alternatingly. Although bilateral movement training mainly describes bilateral upper limb training, recent research has also explored the efficacy of bilateral lower limb training for improving balance and walking in stroke survivors [7,30]. This paper will utilize bilateral movement training to describe bilateral upper and lower limb training protocols. Table 1 summarizes the specific definitions used throughout the paper.

The mechanisms underlying BMT include interlimb cross-transfer effects between the upper and lower limbs, interlimb coupling between the upper and lower limbs, cortical disinhibition, increased recruitment of ipsilateral pathways, and the upregulation of descending commands [24,25,27].

Of particular interest are the significant positive outcomes of the various BMT protocols, such as rhythmic alternating movements used during bilateral arm training with rhythmic auditory cueing (BATRAC) and coupled bilateral training with EMG (Electromyography)-triggered neuromuscular stimulation [8]. These protocols involve training methods that engage both limbs simultaneously, aiming to enhance motor function and coordination. Studies have shown that training with BATRAC, coupled with bilateral and active stimulation protocols, can substantially improve motor capabilities, particularly in individuals with stroke [8]. Functional multichannel neuromuscular electrostimulation has been highlighted as a practical approach to induce specific movements and improve upper extremity function in stroke patients [34].

Moreover, research by Cauraugh and Kim indicated that coupled motor recovery protocols incorporating EMG-triggered neuromuscular stimulation and bilateral movement training resulted in superior motor improvement compared to unilateral training methods. This suggests that combining these techniques can lead to better outcomes in stroke rehabilitation. Additionally, a meta-analysis reported that combining EMG-triggered neuromuscular stimulation with bilateral training significantly enhanced upper limb function in patients with chronic stroke [32,35].

Furthermore, the involvement of the reticulospinal system in neural coupling during bilateral hand movements has been investigated, indicating the importance of brainstem motor centers in coordinating such movements [36]. This neural coordination is crucial for optimizing motor recovery and functional outcomes in individuals post-stroke. Additionally, studies have shown that early initiation of FES-assisted gait training in stroke survivors can lead to improved functional outcomes and reduced therapy duration [37].

In summary, the integration of various BMT protocols, including BATRAC and coupled bilateral training with EMG-triggered neuromuscular stimulation, has shown promise in promoting motor recovery and functional improvements in individuals with stroke. These protocols target bilateral coordination, muscle activation, and neural coupling, all of which are critical for enhancing motor function after stroke.

Other research indicates that BMT can improve upper limb function in patients with chronic stroke [29,38]. While both bilateral and unilateral training offer benefits, bilateral training may be superior for enhancing shoulder motion and upper limb strength [29,39]. Conversely, unilateral training may improve unilateral jumping performance and activities of daily living [39,40]. Both methods appear equally effective for lower limb function and horizontal movement performance [41]. Bilateral arm training has significantly improved motor impairment, as assessed by the Fugl-Meyer Assessment [42]. The choice between bilateral and unilateral training should align with specific rehabilitation goals, with a combined approach potentially offering the most comprehensive benefits and providing reassurance about the adaptability of BMT in stroke rehabilitation [2,7].

Bilateral movement training simultaneously engages both the affected and unaffected limbs, promoting motor function and recovery after a stroke. Over the past decade, studies have highlighted the effectiveness of BMT in stroke rehabilitation, demonstrating significant upper limb recovery [43,44]. BMT is associated with increased activation of the non-affected motor cortex during movements, reflecting its impact on neural processes [45]. Robotic systems have also gained attention for their potential to enhance post-stroke motor rehabilitation [46].

The benefits of BMT extend to various aspects of stroke recovery, facilitating functional motor recovery of the upper extremities [47], promoting rapid improvements in motor performance, and enhancing movement quality after an ischemic infarct in the motor cortex [48]. Bilateral priming has been shown to improve the efficacy of movement therapy, particularly for patients with low motor function following stroke [49]. The significant improvements in motor performance, especially when combined with general occupational therapy, should encourage and motivate healthcare professionals and stroke patients about the potential of BMT in stroke rehabilitation [48].

Additionally, sequencing bilateral and unilateral task-oriented training has been suggested to enhance gains in arm and hand function in individuals with moderate to severe paresis post-stroke [50]. This sequential combination significantly increases motor cortex activation during hand movement, highlighting its potential to improve functional outcomes [50].

While some studies have shown promising results in using bilateral movement training to expedite progress in upper limb recovery post-stroke [51], there is also critical research that questions the efficacy of this approach. A study by Syed et al. found that while bilateral extremity training improved the amount of arm usage, the quality of movement did not show significant improvement. This suggests that while bilateral training may increase the overall use of the arms, it may not necessarily enhance the quality of movement, which is crucial for functional recovery [52].

Moreover, Shih et al. highlighted inconsistent results in longitudinal studies regarding bilateral movement rehabilitation approaches such as BATRAC and bilateral arm training (BBT) [53]. This inconsistency in outcomes raises concerns about the reliability and effectiveness of bilateral training methods. Additionally, Dembele et al. conducted a meta-analysis comparing the effects of bilateral and unilateral training in (sub)acute stroke [43]. They found that integrating high-dosage bilateral movements may not significantly improve the quality of upper limb recovery after stroke.

Furthermore, while some research has suggested that bilateral training can improve motor recovery and functional laterality [2], other studies have raised doubts about the extent of these benefits. For instance, Wang et al. indicated that the effect of bilateral training on subsequent unilateral performance is robust but may not be sensitive to the context of bilateral training [54]. This suggests that while bilateral training may have some transfer effects on unilateral performance, the specificity and magnitude of these effects may vary. Moreover, Wu et al. found that distributed constraint-induced therapy, which focuses exclusively on unilateral training, resulted in similar improvements in movement smoothness compared to bilateral arm training [55]. This challenges the notion that bilateral training is superior to unilateral training in all motor control and recovery aspects. Additionally, Langan et al. suggested that the influence of the task itself plays a significant role in interlimb coordination, indicating that the type of movement involved in bilateral training protocols may impact their effectiveness [56]. Table 2 presents the interventions and their underlying neurophysiological mechanisms discussed in this review.

#### 3.1.1. Bilateral Arm Training

Bilateral upper extremity movement training, primarily focusing on arm and hand exercises, has been extensively studied for its effectiveness in enhancing motor activity and function in individuals with hemiplegia or stroke. This training emphasizes synchronizing and coordinating movements in both limbs simultaneously [43]. It typically involves repetitive practice of identical bilateral arm movements in symmetrical or alternating patterns, as well as bimanual training where both limbs perform different tasks [43].

Over the last two decades, bilateral upper extremity movement training has emerged as an effective intervention for stroke rehabilitation. Numerous studies have demonstrated its efficacy in improving motor function and recovery [2,8,19,28,29,50,58,87,88]. Various bilateral upper extremity training protocols, including alternating hand movements, movements preceded by bilateral robotic motor priming, meaningful daily task training, and error-augmented task training, have been investigated, further validating the approach [29,58,62,68,89].

Recent research on bilateral arm training (BAT) has expanded our understanding of effective interventions for post-stroke rehabilitation [89]. Studies have incorporated bilateral hand movement training [63,64,68,69] and have focused on the effects of bilateral arm interventions on shoulder function [29], coordination, and trajectory control [57,70]. Combining bilateral upper extremity training with other therapies, such as occupational therapy [89] or bilateral robotic movement priming, has shown additional benefits.

Research by Bruyneel suggests that bilateral training may surpass traditional unilateral methods, as it more closely mimics real-life tasks. Thus, it reinforces movement patterns and strengthens ecological validity within rehabilitation programs [57,90].

The inclusion of sensory feedback within BAT has also emerged as an influential factor in optimizing recovery outcomes. Han and Kim highlight how sensory feedback mechanisms—like visual and auditory cues—can improve patients’ engagement and comprehension of their movements, enhancing motor learning. Their findings align with emerging practices that leverage technology to enrich sensory feedback, presenting a promising avenue for rehabilitation protocols. Such approaches could enable patients to understand and adjust their movements better, facilitating a deeper integration of motor skills [29,91].

Kim et al. investigate the neurophysiological processes underlying bilateral arm training (BAT), presenting evidence that bilateral training may influence neuroplasticity, a crucial aspect of motor recovery following neurological injury. BAT appears to induce changes in brain activity and connectivity in regions responsible for motor control, suggesting that it may profoundly impact the brain’s ability to rewire and adapt after injury. Kim et al.’s study emphasizes the importance of examining how distinct rehabilitation modalities can impact neural networks, thereby guiding targeted and effective interventions in motor recovery [61].

BAT’s adaptability extends to younger populations, as shown by Kumagai, who investigates its application in pediatric patients with hemiparesis. Kumagai et al.’s findings reveal that BAT can facilitate significant improvements in motor function and coordination in children, with benefits that parallel those seen in adult populations. Early intervention appears particularly advantageous in neurodevelopment, as BAT’s repetitive, bilateral movements may help solidify motor pathways during a critical period of growth and learning [20,62].

Extending the scope of BAT applications, Kaupp et al. investigate the impact of bilateral arm training in pediatric populations, particularly in children with hemiparesis. The research indicates that BAT yields significant improvements in motor function and coordination in children, mirroring the positive results observed in adults. Kaupp et al.’s study advocates for early intervention, proposing that BAT may positively influence developmental outcomes in children with motor impairments. This perspective emphasizes the importance of tailoring BAT protocols to be age-appropriate, acknowledging the distinct neurodevelopmental needs of younger patients to optimize their rehabilitative potential [24,87].

Research has highlighted the significance of task-specific versus non-task-specific BAT. Some experimental studies have found that autonomy in task control during bilateral upper extremity movement training has a significant positive impact on outcomes [68]. Bilateral upper arm movement training can be categorized into task-oriented training, which focuses on goal-directed movements, and movement-oriented training, enhancing sensorimotor abilities such as speed, accuracy, and endurance [61,63].

In exploring the efficacy of bilateral arm training (BAT) in stroke rehabilitation, Lee demonstrates how BAT can facilitate substantial improvements in motor function, particularly in the functional use of the affected arm. This study underscores that engaging both arms simultaneously during rehabilitation strengthens the affected limb and promotes crucial interlimb coordination, enhancing the patient’s ability to perform everyday tasks. Lee’s findings align with theories of bilateral training by suggesting that engaging both arms capitalizes on the neural connections between the brain’s hemispheres, thereby supporting the functional recovery of the impaired limb [54,89].

Task-specific training involves intensive practice of actions or functional tasks relevant and significant to the individual’s daily life, promoting neuroplasticity, motor learning, and improved functional reorganization. Studies suggest that engaging in task-specific actions leads to better rehabilitation outcomes than non-task-specific approaches, highlighting the critical role of tailored and meaningful task training in optimizing recovery and promoting neural reorganization in individuals with stroke. Repetition alone without functional meaning is insufficient to produce meaningful improvements in rehabilitation [92].

Task-specific training is recommended internationally in stroke rehabilitation guidelines and involves intensive practice of actions or functional tasks [20]. Rehabilitation therapies that involve task-specific actions often have more effective outcomes than traditional, non-task-specific rehabilitation therapies [20]. This is supported by a systematic review highlighting the benefits of task-oriented training for improving functional outcomes in stroke patients, indicating that such training is more effective than conventional therapies [20].

Moreover, the integration of task-specific training into rehabilitation protocols has been shown to enhance motor function recovery, particularly in the upper limbs, by promoting neuroplasticity and facilitating the relearning of motor skills [93]. The evidence suggests that engaging patients in meaningful and functional tasks improves their motor abilities and enhances their overall quality of life [94]. This aligns with findings that emphasize the importance of personalized and intensive practice in rehabilitation settings, which can significantly improve daily activities and independence for stroke survivors [93,94].

Studies have demonstrated the feasibility of delivering hundreds of repetitions of task-specific training in one-hour therapy sessions, leading to improvements in secondary measures of activity and participation [95]. Activity-Based Restorative Therapies (ABRTs) involve repetitive task-specific training using weight-bearing and external facilitation of neuromuscular activation [96]. Technology, such as virtual reality-based therapy, offers advantages in rehabilitation by maximizing variables that align with neuroplastic processes necessary for stroke rehabilitation, including massed practice, repetition, task specificity, and meaningful tasks [76].

Task-specific training within bilateral upper extremity movement training is crucial for optimizing recovery and promoting neural reorganization in stroke survivors. Task-specific training involves intensive practice of actions or functional tasks relevant to daily life, resulting in better rehabilitation outcomes than non-task-specific approaches. For instance, Cunningham et al. emphasize that task-specific training is recommended in stroke rehabilitation guidelines and has been shown to improve upper limb function significantly through repetitive task training [97]. This aligns with findings from Khallaf, who noted that task-specific training enhances trunk control and balance, essential for daily post-stroke activities [98].

Moreover, task-specific actions promote neuroplasticity, motor learning, and improved functional reorganization. Research indicates that meaningful and tailored task training is vital for effective stroke rehabilitation. For example, Grefkes and Fink discuss how training-based interventions enhance functional recovery and neural plasticity, underscoring the importance of targeted rehabilitation strategies [99]. Similarly, the work of Demers et al. highlights the neural plasticity changes associated with task-specific training, suggesting that such interventions can lead to significant cortical reorganization in patients with chronic stroke [100].

Furthermore, the delivery of hundreds of repetitions of task-specific training in therapy sessions has improved secondary measures of activity and participation. This is supported by findings from Iqbal et al., which demonstrate that task-oriented training significantly enhances balance and performance in activities of daily living in stroke patients [101]. Additionally, the systematic review by Chiaramonte et al. reinforces the notion that task-specific training is essential for improving balance control and reducing fall risk, particularly during dual-tasking scenarios [102]. These studies collectively emphasize the importance of task specificity in rehabilitation, highlighting its role in promoting recovery and improving the quality of life for stroke survivors.

Integrating bilateral upper extremity training with other therapies, such as occupational therapy or bilateral robotic movement priming, has shown additional benefits in stroke rehabilitation [103].

#### 3.1.2. Bilateral Arm Training Plus Sensory Enhancement

Recent research underscores the growing potential of bilateral arm training (BAT) combined with sensory enhancement as a powerful approach in stroke rehabilitation. Integrating sensory enhancement modalities, such as visual, auditory, or tactile feedback, refines BAT by providing real-time information that enhances movement accuracy and facilitates motor learning. Studies indicate that this sensory feedback amplifies the engagement of motor and sensory pathways, facilitating adaptive changes in the brain that improve coordination and function in the affected arm. For instance, Wang et al. highlight that the neural mechanisms underlying motor learning significantly overlap between bilateral and unilateral training, suggesting that BAT can facilitate functional recovery of the paretic arm in stroke patients with hemiparesis [54]. Furthermore, Chuang et al. demonstrate that bilateral arm training, when combined with neuromuscular electrical stimulation, can lead to improved arm function and reduced shoulder pain in patients with hemiplegia, indicating the efficacy of this approach in enhancing rehabilitation outcomes [104].

The role of sensory feedback in motor learning is further supported by the findings of Huang et al., who report that both bilateral and unilateral training can induce changes in cortical sensorimotor maps, thereby improving motor function post-stroke [69].

Robotic-assisted therapy has been shown to provide intensive and repetitive training, which is crucial for forming new neural pathways in the brain [105]. When robotic-assisted therapy is complemented by mirror therapy, which utilizes visual illusions to promote movement in the affected limb by reflecting the movements of the unaffected limb, this technique has been shown to enhance motor recovery by engaging the brain’s visual and motor systems, thereby facilitating interhemispheric communication [69,106]. Furthermore, the integration of these advanced rehabilitation techniques can lead to significant improvements in motor function and neural plasticity, as they encourage the brain to reorganize and adapt following injury [107,108].

Sun and Zehr [77] demonstrated that sensory enhancement can amplify interlimb cutaneous reflexes in wrist extensor muscles. This finding aligns with the impact of sensory enhancement on the success of bilateral arm training (BAT). Sensory enhancement can amplify interlimb cutaneous reflexes in wrist extensor muscles, as demonstrated by Sun and Zehr [77]. This finding aligns with the impact of sensory enhancement on the success of bilateral arm training (BAT). The amplification of interlimb reflexes is particularly relevant in cooperative bimanual tasks, where stronger reflexes are observed when both arms are dynamically coupled, compared to performing independent static tasks. This suggests that shared cutaneous input during bilateral movements can enhance motor control and coordination, which are crucial for effective rehabilitation strategies [77]. Furthermore, bilateral arm training can induce the concurrent activation of neural pathways, leading to improved motor control in the affected limb through mechanisms such as cortical disinhibition and enhanced interhemispheric communication, as noted by Chuang et al. [104]. These findings support the notion that sensory enhancement facilitates reflexive responses and contributes to the overall efficacy of bilateral training interventions in rehabilitation settings.

Song et al. focused on the role of sensory enhancement in facilitating motor performance, examining the influence of sensory feedback—particularly visual and tactile—on motor task accuracy and speed [66]. Their findings indicated that sensory feedback substantially improved both aspects of performance, likely by providing additional sensory information to aid motor planning and execution. This supports the notion that sensory input is fundamental to motor control, as it allows for real-time adjustments and fine-tuning of movements, making it a critical component in motor rehabilitation [109].

Building on these findings, Rodrigues et al. [65] investigated the integration of sensory feedback into motor training protocols, finding that enhanced sensory modalities during training contributed to improved motor learning and performance. Participants who received augmented sensory feedback exhibited more significant gains than those who trained without this added feedback, suggesting that sensory enhancement could be a valuable tool for rehabilitation and skill acquisition. These results underscore the importance of sensory input in optimizing motor function, reinforcing the value of sensory-integrated strategies in clinical settings [110].

Unfortunately, one of the primary methodological challenges across these studies is the inconsistency in BAT protocols, which complicates direct comparisons and limits generalizability. Van Delden et al. demonstrated the efficacy of BAT for individuals with hemiparesis. However, the specific training regimens varied considerably across studies, making it difficult to understand the exact impact of BAT consistently [67]. Similarly, Song et al. [66] examined the effects of task-oriented versus repetitive BAT but did not achieve a clear consensus regarding optimal training duration or intensity. The absence of standardized protocols in BAT research underscores the need for future studies to develop uniform guidelines, thereby enhancing the comparability and reliability of findings.

In addition to methodological challenges, the studies primarily address stroke patients but often lack diversity in stroke-related conditions and population characteristics. For instance, Rodrigues et al. emphasized the role of sensory feedback in enhancing motor learning; however, the findings were primarily limited to specific stroke subpopulations [65].

#### 3.1.3. Bilateral Arm Training and Robotics

Robotic solutions in stroke rehabilitation offer several benefits, including providing repetitive movements, objective measurements of functional improvements, and adapting training tasks to meet patient needs [111]. Robotic systems can also assist in re-educating balance, walking, and improving lower limb function in post-stroke survivors [30]. Additionally, robotic devices can be crucial in functional hand rehabilitation after a stroke, providing training and assistance with daily activities [112].

Robotic devices in bilateral training, such as the Mirror Image Movement Enabler (MIME), have shown promise in providing shoulder and elbow neurorehabilitation in subacute stroke patients [113]. These robotic devices offer bilateral training modes that can enhance the effectiveness of rehabilitation by providing repetitive and consistent training movements [114].

The control systems integrated into these devices enable precise movements and can provide electrical stimulation to specific muscle groups, facilitating plasticity and recovery in post-stroke patients [114]. The design of robotic exoskeletons is crucial in delivering intensive, personalized, and cost-effective physiotherapy, which is essential for optimizing rehabilitation outcomes, especially in stroke survivors [115].

Moreover, comparing active and active-assistive robotic rehabilitation groups in stroke survivors highlights the importance of robotic interventions in delivering consistent and structured training sessions, which are essential for promoting recovery [116].

Robotic devices have diverse benefits. Their ability to increase motivation, adapt training tasks based on individual needs, collect data for monitoring progress, ensure patient safety, and enable intensive repetitive practice underscores their significance in enhancing rehabilitation effectiveness [117]. The development of wearable robotic devices for in-bed acute stroke rehabilitation underlines the potential for robotics in delivering targeted and accessible rehabilitation interventions [118].

Integrating bilateral arm training (BAT) and robotic assistance in stroke rehabilitation has gained considerable traction, as evidenced by the research contributions of AbdollahI et al., Huang et al., and Li et al. [68,69,119]. Together, their studies underscore the promising role of robotic-assisted therapies in enhancing motor function, coordination, and overall recovery in stroke patients.

Compared to traditional therapist-led rehabilitation, Huang et al. demonstrated that robotic assistance can significantly improve motor control and functional performance in stroke patients, particularly in tasks requiring bilateral coordination. This led to additional increases in independence in daily activities [69]. The consistency and intensity of training provided by robotics emerge as a key advantage over conventional therapy, which may vary in quality and intensity due to human limitations [120].

Li et al.’s research takes a novel approach by examining the impact of robotic priming techniques combined with BAT [119]. Their findings suggest that integrating robotic priming with task-oriented therapies effectively reduces motor impairments and encourages the functional use of the affected arm in everyday tasks [121].

#### 3.1.4. Bilateral Arm Training and Virtual Reality/Computer Guidance

When combined with modified constraint-induced movement therapy, virtual reality training is a practical approach for recovering upper extremity function in patients with acute stroke [122]. Furthermore, virtual reality technology (VR) has been utilized in various fields, including dance movement analysis, music integration training for sports, and balance training for the elderly, demonstrating its versatility and effectiveness in movement training [119,123,124].

Jayasinghe et al.’s paper on bilateral arm training (BAT) integrated with virtual reality (VR) and video guidance found a significant increase in patient motivation and engagement in rehabilitation [70]. The immersive nature of VR creates an interactive environment that encourages active participation, which is crucial given the established link between patient motivation and positive rehabilitation outcomes [79]. Furthermore, VR’s interactive and enjoyable aspects are suggested to enhance the retention of motor skills practiced during therapy, facilitating repeated movement practice essential for neuroplasticity and motor recovery [26]. By fostering a stimulating atmosphere, VR-based BAT could thus play a critical role in improving patient adherence and maximizing the therapeutic benefits of rehabilitation.

Jayasinghe et al. also highlight the advantages of video guidance (VG) with BAT, providing real-time feedback for motor learning. Immediate visual feedback enables patients to observe and correct their movement patterns, thereby supporting motor learning through improved accuracy and coordination over time [125]. This feedback mechanism is particularly relevant in stroke rehabilitation, where patients frequently encounter challenges with coordination and movement execution due to motor impairments. VG also aids in monitoring and adjusting training protocols based on individual needs and progress.

### 3.2. Bilateral Leg Training

Walking therapy is one of the most widely used forms of post-stroke rehabilitation; however, it is often one of the only bilateral leg therapies used during the initial rehabilitation period [126]. Research shows that early and intensive walking training can significantly enhance stroke survivors’ motor function and mobility recovery [127].

Over the past decade, walking therapy has been recognized as a crucial component in post-stroke rehabilitation. Studies have highlighted the importance of whole-body activities, such as walking, in enhancing recovery after a stroke [128]. Research has shown that interventions focusing on walking can significantly improve walking speed, balance ability, and overall functional recovery in stroke patients [129]. Moreover, varying doses of higher-intensity, task-specific walking-related interventions have been investigated to enhance walking recovery, physical function, cognition, and overall well-being after stroke [130]. The findings from Wonsetler and Bowden emphasize that walking endurance is vital for home and community walking activities after a stroke, which aligns with the notion that walking therapy is integral to rehabilitation [128]. Furthermore, Khan et al. demonstrate that task-oriented walking interventions can significantly aid in the early recovery of stroke patients, reinforcing the effectiveness of walking-focused rehabilitation strategies [16]. These studies collectively underscore the importance of structured walking interventions in enhancing recovery outcomes for stroke survivors.

Recent findings suggest that targeted interventions can enhance lower limb motor function and address early ankle dorsiflexion dysfunction, a crucial factor in mobility and balance [131]. Furthermore, the efficacy of bilateral therapy, primarily through lower limb strengthening exercises, has been demonstrated to effectively promote balance in patients with hemiparetic stroke. This approach highlights the benefits of bilateral training in enhancing rehabilitation outcomes for individuals recovering from stroke [30,131]. The benefits of bilateral training are further supported by studies that highlight its role in improving functional reach and balance scores among stroke survivors. Jeon and Hwang’s randomized controlled trial illustrated that patients engaging in bilateral lower limb strengthening exercises exhibited significantly improved balance and walking capabilities compared to those undergoing unilateral training [131]. This finding aligns with the broader literature, which suggests that bilateral training not only aids in restoring motor function but also plays a crucial role in improving overall balance and mobility in post-stroke rehabilitation [30].

Walking therapy is a fundamental aspect of post-stroke recovery; however, there are scenarios where alternative or supplementary interventions may be more appropriate based on individual needs. For instance, in cases where individuals with stroke have significant balance impairments, incorporating backward walking training alongside conventional therapy has been shown to enhance balance and functional outcomes [132].

Moreover, for stroke survivors with cognitive deficits that impact their ability to engage effectively in walking therapy, virtual reality training with cognitive load has been proposed as a beneficial approach to improving walking function [133].

Studies have explored the efficacy of exoskeleton-based physical therapy programs and functional electrical stimulation gait training in improving gait performance, walking speed, balance, and overall activity post-stroke [134]. Functional electrical stimulation therapy has been shown to significantly improve walking ability and motor recovery in chronic stroke patients when combined with conventional treatment [135]. Robotic exoskeletons for overground walking have shown promise in enhancing functional outcomes, such as increased walking speed, which is a strong predictor of independent community ambulation [136]. For non-ambulatory stroke patients or those with severe mobility limitations, early rehabilitation programs utilizing exoskeleton-based physical therapy have been recommended to be goal-oriented, repetitive, and task-specific to optimize gains in mobility and walking [134]. Furthermore, in instances where stroke survivors have reached a plateau in their recovery despite ongoing rehabilitation efforts, gait training with wearable robotic devices has led to further improvements in walking ability [137].

Ardestani et al. and Jo found that BLT (bilateral leg training) leads to notable improvements in muscle strength and functional performance compared to unilateral training [85,86]. Participants engaging in BLT demonstrated significantly more significant strength gains attributed to the simultaneous activation of both legs during exercises, which enhances neuromuscular adaptations and benefits overall lower limb functionality [138]. This bilateral approach supports more balanced strength development across both legs, reducing the risk of compensatory movement patterns that could lead to further injury—a critical consideration for individuals recovering from injuries or surgeries [139].

Furthermore, Ardestani and Jo emphasize incorporating functional tasks into BLT. Their findings suggest that combining BLT with task-oriented activities, such as squats or step exercises, enhances performance in daily living tasks. This is particularly relevant in rehabilitation, as improved functional performance translates to better mobility and independence in daily life [140]. The researchers argue that integrating functional movements helps build strength, improves coordination, and enhances balance, which are vital for maintaining overall mobility and independence.

Additionally, bilateral therapy involving lower limb strengthening exercises has effectively promoted balance in patients with hemiparetic stroke, further supporting the benefits of bilateral training in stroke rehabilitation [7,131].

#### Bilateral Leg Training and Sensory Enhancement

Transcutaneous electrical nerve stimulation (TENS) has emerged as a significant adjunctive intervention for enhancing various aspects of rehabilitation in individuals who have suffered strokes, including improvements in paretic lower-limb muscle strength, walking speed, balance performance, and overall functional mobility. The therapeutic effects of TENS are mediated through both peripheral and central mechanisms [141]. From a peripheral perspective, TENS applied to paretic limbs has been shown to influence spinal reflex pathways, potentially enhancing motor control. However, the specific effects on H-reflex amplitude and latency in chronic stroke patients are not adequately supported by the cited reference [142], focusing on analgesic mechanisms rather than motor control. Therefore, this claim should be revised to reflect the general understanding of TENS effects without specific unsupported details. Centrally, TENS has been demonstrated to affect cortical processes. For instance, a study found that a single session of electrical stimulation on a paretic hand resulted in changes in cortical excitability and connectivity during thumb contractions on the affected side [143]. This finding highlights the potential of TENS to enhance cortical excitability and connectivity, which are essential for motor recovery following a stroke.

Moreover, a regimen of TENS combined with task-oriented training (TOT) has been shown to yield improvements in lower-limb muscle strength and walking performance compared to placebo-TENS combined with TOT in individuals with chronic stroke [141]. This suggests that integrating TENS with structured rehabilitation protocols can significantly enhance recovery outcomes in stroke patients. Additionally, the research by Lim and Madhavan on non-paretic leg movements facilitating cortical drive to the paretic leg provides further insights into the efficacy of combined sensory and motor interventions for improving motor function in stroke patients [141]. This approach aligns with the understanding that enhancing sensory feedback can facilitate motor recovery, as sensory inputs play a critical role in motor learning and rehabilitation.

The research by Kwong et al. on bilateral leg training (BLT) combined with sensory enhancement training provides valuable insights into its efficacy in improving motor function and sensory integration, among other benefits, in individuals with lower limb impairments [73]. However, several notable research gaps remain, highlighting areas where further investigation is essential.

A primary limitation of Kwong et al.’s study is the lack of investigation into the long-term effects of BLT combined with sensory enhancement on functional independence and quality of life. While the findings indicate immediate gains in motor function and sensory integration, little is known about the sustainability of these improvements. Longitudinal studies that follow participants over extended periods are needed to determine the durability of these benefits and their influence on overall functional capabilities in daily life [73].

Furthermore, there is a need for a more detailed examination of the neurophysiological mechanisms underlying the benefits of this combined training approach. While Kwong et al. report improvements in motor and sensory functions, their study does not examine the neural adaptations that may underlie these effects. Employing neuroimaging techniques could help clarify how BLT with sensory enhancement influences brain plasticity and interhemispheric communication [73]. This is especially relevant for individuals with neurological impairments who may benefit from targeted rehabilitation strategies.

Lastly, Kwong et al.’s study, like many studies covering bilateral arm and/or leg training, does not fully consider the psychosocial aspects of rehabilitation, which can be critical in influencing recovery trajectories [73].

### 3.3. Bilateral Arm and Leg Training

Recent studies have increasingly focused on using bilateral arm and bilateral leg training in stroke rehabilitation to enhance motor recovery and functional outcomes in individuals post-stroke. By integrating bilateral arm and leg training, researchers aim to target comprehensive motor recovery and functional improvements in individuals with stroke [24].

Studies have highlighted the effectiveness of bilateral arm training in improving upper extremity function post-stroke, emphasizing its role in facilitating motor recovery and enhancing daily activities [144]. Additionally, incorporating bilateral leg training has improved walking ability and enhanced neurophysiological integrity in patients with chronic stroke, indicating the potential benefits of combining bilateral arm and leg interventions [24]. This integrated approach aims to address both upper and lower limb impairments commonly observed in stroke survivors, providing a holistic rehabilitation strategy.

Arya et al. [15] provide significant evidence on the effectiveness of combined bilateral arm and leg training in enhancing motor function and rehabilitation outcomes for individuals with mobility impairments. Their study highlights the benefits of integrating upper and lower limb training, which promotes a more holistic approach to functional recovery.

One of Arya et al.’s findings is that combined bilateral arm and leg training yields more substantial improvements in overall motor function than unilateral training. Participants who engaged in this combined training regimen demonstrated increased strength and coordination across both arms and legs—an essential factor for performing activities of daily living. This outcome supports existing research suggesting that bilateral training facilitates cross-education effects, whereby training one limb induces strength gains in the untrained limb due to neural adaptations [42,74]. Arya et al. argue that this approach enhances strength and builds the inter-limb coordination needed for complex, simultaneous movements involving both arms and legs.

Further, Arya et al. emphasize the role of combined training in fostering neuroplasticity [15]. The study demonstrates that simultaneous activation of the upper and lower limbs in rehabilitation can stimulate the brain’s capacity for reorganization and adaptation. This is especially valuable for individuals with neurological impairments, such as those resulting from a stroke. The authors propose that engaging multiple muscle groups together may expand the cortical representation of the trained limbs, leading to improved motor control and functional recovery [15]. This neuroplastic response is critical for refining movement quality and mitigating fall risks, a common challenge for individuals with mobility impairments.

The research also highlights the value of incorporating task-oriented training within the combined regimen. Arya et al. found that including functional tasks, such as reaching and stepping, during sessions significantly enhanced skill transfer to real-world activities. This approach strengthened and coordinated the limbs, promoting greater independence in daily life—a key goal of rehabilitation [15]. Arya et al. advocate for integrating functional movement in rehabilitation programs to ensure that gains in strength and coordination translate into meaningful improvements in quality of life for individuals facing mobility challenges [15].

In the paper by Arya et al. on combined rhythmic arm and leg training, several significant research gaps can be identified that could inform future studies and enhance the understanding of this rehabilitation approach [15]. These gaps highlight areas where further investigation could improve outcomes for individuals undergoing rehabilitation.

A significant research gap exists in the need for a more comprehensive understanding of the long-term effects of combined rhythmic arm and leg training on functional outcomes. While Arya et al. demonstrate immediate improvements in motor performance, the sustainability of these benefits, particularly regarding functional independence in daily activities, remains underexplored [15].

### 3.4. Bilateral Movement Priming

Bilateral motor priming (BMP) is a form of neuromodulation methodology rather than an independent therapeutic intervention. This approach can involve mirror-image bilateral wrist movements facilitated by a device with mechanical components that ensure the synchronized movement of the less affected and affected hands [74,145]. Unlike bilateral training, which focuses on bilateral movements, bilateral symmetrical actions in BMP are classified as a neuromodulation technique. Through a case–control investigation, it was observed that BMP conducted before Wii-based therapy yielded enhanced therapeutic outcomes for post-stroke patients, including those with severe impairments [47].

Additionally, studies have explored the use of bilateral upper extremity motor priming (BUMP) combined with task-specific training for severe, chronic upper limb hemiparesis, highlighting the potential benefits of this approach [74]. Furthermore, the effectiveness of bilateral motor priming has been linked to its ability to increase corticomotor excitability in the primary motor cortex [84]. This increase in corticomotor excitability may enhance motor learning and recovery in individuals undergoing motor rehabilitation following conditions such as stroke [85].

Moreover, combining high-dose therapy, bilateral motor priming, and vagus nerve stimulation has been proposed as a comprehensive approach to treating the hemiparetic upper limb in chronic stroke survivors, emphasizing the potential of integrating different rehabilitation techniques for enhanced recovery [146]. In neurorehabilitation, understanding various motor priming paradigms and their underlying neural mechanisms is crucial for optimizing therapeutic interventions [1]. Studies have also highlighted the importance of movement-based priming in post-stroke rehabilitation, such as continuous wrist flexion and extension through low-tech devices, showcasing the practical applications of neuromodulation techniques in clinical settings [147]. Additionally, research has explored the effects of robotic priming combined with mirror therapy and bilateral upper limb training in stroke survivors, indicating the potential benefits of hybrid therapies in rehabilitation [121].

## 4. The Underpinning Neurophysiological Mechanisms of Bilateral Movement Training and Interlimb Coupling

Interhemispheric dynamics and cortical reorganization: Bilateral Movement Training (BMT) is an increasingly utilized strategy in stroke rehabilitation due to its robust capacity to engage the nervous system comprehensively and synchronized. After a cerebrovascular accident, the lesioned hemisphere commonly exhibits depressed excitability, while the contralesional (unaffected) side may exert excess inhibitory control via transcallosal pathways [148,149]. This imbalance often limits motor recovery for the paretic limb. BMT counteracts this phenomenon by activating both hemispheres simultaneously, promoting symmetrical engagement of the primary motor cortex (M1), supplementary motor areas (SMAs), premotor cortices (PMCs), and associated sensorimotor networks. Over time, bilateral stimulation enhances interhemispheric coherence and reduces maladaptive transcallosal inhibition [27,145], creating a cortical environment more conducive to remapping and functional restoration.

From a neurobiological perspective, cortical reorganization in BMT is driven mainly by Hebbian mechanisms of synaptic plasticity. In the case of motor recovery, these mechanisms operate through long-term potentiation (LTP)—where repeated, the simultaneous activity of pre- and postsynaptic neurons leads to strengthened synaptic connections—and through associated increases in neurotrophic factors such as brain-derived neurotrophic factor (BDNF) and nerve growth factor (NGF) [148,149,150]. These biomolecules facilitate synaptogenesis, dendritic spine remodeling, and axonal sprouting, enabling the ipsilesional motor cortex to reestablish or compensate for lost motor functions. In addition, EEG and multimodal studies corroborate that contralesional motor cortex activation can support motor performance, particularly in the early phases of recovery, by serving as a provisional “backup” system [150]. Metrics such as event-related desynchronization (ERD) and coherence have emerged as potential biomarkers, helping clinicians predict a patient’s responsiveness to bilateral interventions [151,152]. This line of evidence suggests that BMT fosters a more favorable cortical equilibrium, allowing both hemispheres to work jointly rather than in a competitive or inhibitory fashion.

Crucially, interlimb coupling shapes how these bilateral cortical reorganizations take place. When both limbs are engaged, the sensorimotor representations in each hemisphere synchronize not only in terms of excitatory–inhibitory balance but also in the temporal coordination of neural firing. This synchronized engagement of sensorimotor maps is critical when stroke-related lesions impair one hemisphere. The contralesional side may “assist” via interlimb coupling, helping the affected hemisphere reorganize more effectively [36,153].

Sensorimotor synchronization and rhythmic entrainment: Beyond enhancing cortical balance, BMT leverages the human brain’s inherent capacity to synchronize with externally provided rhythms. Sensorimotor synchronization represents the alignment of internally generated movement commands with rhythmic cues, such as metronomes or patterned auditory stimuli [154,155]. The phenomenon is underpinned by what is sometimes termed “rhythmic attentional sampling”, a dynamic whereby attentional resources in sensorimotor cortices are allocated in discrete time windows, reducing interference between competing motor tasks [156]. EEG experiments show that these rhythms manifest in oscillatory activity within alpha, beta, and gamma bands, enabling the motor system to precisely time force outputs and coordinate multi-joint actions [157].

A critical element of this mechanism involves subcortical loops, particularly basal ganglia circuits, which coordinate habitual and repetitive movement sequences [154,155]. After stroke, basal ganglia dysfunction often yields deficits in movement initiation and timing, compounding the difficulties of motor recovery. By engaging auditory or other sensory cues to entrain movement, BMT can partially restore the integrity of these basal ganglia–thalamo–cortical loops, thereby normalizing temporal aspects of motor output [158,159]. Rhythmic strategies also facilitate the consolidation of newly acquired or relearned skills, complementing Hebbian plasticity in cortical areas and cementing functional improvements in tasks of daily living.

Regarding interlimb coupling, rhythmic entrainment encourages the limbs to move in complementary cycles, reinforcing bilateral coordination patterns at both the cortical and subcortical levels. The synchronicity of auditory cues and limb movement unites the sensorimotor systems of each limb, compelling them to “lock in” to one another. This process can be particularly valuable for individuals who favor the unaffected limb since the rhythmic coupling forces the paretic limb to keep pace and integrate with a shared motor command stream [26,160,161].

Central Pattern Generators and spinal circuitry: Another core advantage of BMT is its activation of Central Pattern Generators (CPGs)—neural circuits in the spinal cord and brainstem that autonomously generate rhythmic motor outputs, including walking and cycling [162,163,164]. Post-stroke damage to upper motor neurons and descending pathways can impede access to these intrinsic motor networks, leading to atypical gait and loss of functional movement patterns. Rhythmic bilateral limb movements, such as arm or leg cycling, re-engage and “tune” CPG activity by providing symmetric sensory feedback and descending drive from both hemispheres [158,165,166]. Recent work by Klarner and colleagues demonstrates that arm and leg cycling exercises can induce reflex modulation across all four limbs, implicating shared spinal and supraspinal circuits [25,167]. This reinforces that a coordinated bilateral approach provides a potent stimulus for CPGs, enhancing interlimb coordination, gait parameters, and overall locomotive efficiency.

Reflex studies further illustrate how CPGs benefit from bilateral training. Rather than a simple sum of unilateral outputs, BMT evokes bidirectional communication among spinal cord segments, leading to more adaptive neuromuscular responses and improved synchronization in interlimb tasks [168]. This integrated activation likely stems from the facilitation of propriospinal pathways—neural routes bridging cervical and lumbar enlargements—essential for coupling arm and leg movements. By systematically modulating these pathways, BMT helps restore refined motor patterns lost after stroke.

Notably, interlimb coupling at the CPGs and spinal circuitry level often manifests as naturally emerging patterns of reciprocal or synchronous movement. During bilateral cycling, for instance, feedback from one limb can modulate the reflex arcs of the other, highlighting how interlimb coupling can refine overall movement synergy and entrain symmetrical gait cycles [153].

Use-dependent plasticity and sensorimotor integration: BMT’s efficacy also hinges on use-dependent plasticity—the principle that motor practice reshapes cortical and subcortical representations according to task demands. Repetitive, bilateral tasks prime the motor system to refine synaptic maps, increase cortical excitability, and form robust neural representations of new movement sequences [169,170,171]. Volitional exercise paired with precise sensory feedback strengthens connections in sensorimotor circuits, an effect amplified by technologies such as proprioceptive robotics, haptic interfaces, and sensory-augmentation protocols [77,143]. Tactile or proprioceptive inputs act as “anchors,” consolidating motor commands within appropriate cortical or spinal loci.

Studies of stroke survivors reveal that when voluntary motor output is paired with focused somatosensory input—via techniques like cutaneous reflex stimulation, TENS, or proprioceptive tapping—motor cortex reorganization accelerates [141,172,173,174,175]. Neural recordings indicate heightened synaptic efficiency in corticospinal projections and reduced noise in sensorimotor integration, culminating in more accurate and coordinated limb movements. Over time, these sensorimotor adaptations feed into cortical reorganization, establishing a virtuous cycle wherein repetition of each practice reinforces more stable and efficient motor plans.

In interlimb coupling, sensorimotor integration across both sides of the body helps unify bilateral control strategies. For example, symmetrical tactile cues on both arms or legs may synchronize the firing patterns of sensory afferents, prompting the cortex to perceive, process, and execute motor commands in a unified manner. The brain refines its overarching sensorimotor schema by continuously merging proprioceptive feedback from both limbs and encourages greater cooperation between the impaired and unimpaired sides [36,77].

Cerebellar and mirror neuron system modulation: A central subcortical structure implicated in BMT is the cerebellum, traditionally recognized for its roles in fine-tuning, error detection, and the timing of voluntary movements [176,177,178]. During bilateral training, the cerebellum receives extensive proprioceptive and motor efference copy signals, enabling it to recalibrate motor programs and minimize movement errors in real-time. This recalibration extends beyond local cerebellar loops, influencing broader cortico–cerebellar networks responsible for sensorimotor adaptation [179,180]. Recent functional neuroimaging in stroke populations shows that re-engaging these loops through complex bilateral or bimanual tasks can partially restore dynamic balance, gait, and gross motor coordination deficits.

Simultaneously, the mirror neuron system (MNS) comes into play, particularly when BMT is embedded within virtual reality (VR), action observation, or motor imagery protocols [181,182,183]. The MNS is activated by executing and observing movements, facilitating motor learning through imitation-based pathways. By pairing bilateral movement practice with VR-based or observational tasks, stroke survivors can harness these MNS circuits more effectively, boosting plastic changes in sensorimotor cortices. Moreover, immersive training settings tap into reward-related dopaminergic circuits, elevating motivation and adherence [78,79]. Neuromodulatory techniques such as cerebellar transcranial direct current stimulation (tDCS) and cerebellar–motor paired associative stimulation (PAS) can amplify these gains by increasing cerebellar excitability and augmenting cerebello–cortical interplay [184,185].

From the vantage point of interlimb coupling, the cerebellum’s real-time calibration of bilateral limb trajectories underpins smooth and coordinated motion. Simultaneous motor commands to both limbs demand enhanced error detection and correction, which the cerebellum processes by comparing expected and actual sensory feedback. In turn, the MNS supports cross-limb observational learning, wherein the action of one limb (or observation of a healthy limb) can facilitate motor re-mapping in the paretic side through sensorimotor mirroring [26].

Interlimb transfer and cross-education: Another crucial benefit of BMT is interlimb transfer, the phenomenon by which training the unaffected limb produces performance gains in the paretic limb. Often referred to as cross-education, this process arises from bilateral activity in sensorimotor cortices and cross-cortical communication via the corpus callosum [186,187,188]. For example, unilateral practice of the less-affected arm can promote excitatory drive in homologous cortical areas of the opposite hemisphere, supporting improvements in the more affected limb. This mechanism is particularly vital in acute or subacute settings, where the paretic side might be initially unresponsive or painful to move. Clinical tools that evaluate real-time motor learning and transfer effects have been introduced, enabling clinicians to tailor BMT more precisely to each patient’s neural profile [189]. Such individualized approaches optimize the likelihood of functional carryover to daily activities.

Interlimb coupling resonates strongly with these transfer effects. While cross-education typically emphasizes unilateral practice transferring to the affected limb, interlimb coupling underscores how the limbs can coordinate to strengthen shared motor networks simultaneously. When both limbs are engaged, cortical and subcortical areas create or reinforce interlinked motor representations, potentially augmenting the cross-education effect and leading to more substantial functional gains than unilateral protocols alone [36,153,190].

Technological augmentation and multimodal integration: Emerging technologies provide an extra dimension to BMT by enhancing repetition, feedback, and patient engagement. Robotic-assisted BMT, for instance, allows precise control of movement trajectories, speeds, and force outputs, enabling stroke survivors to practice symmetrical motor tasks at high intensities [42,69,160]. Meanwhile, EEG-based brain–computer interfaces (BCIs) detect cortical signals reflecting movement intention or motor imagery; these signals control exoskeletons or virtual avatars in real-time [52,143]. Brain-driven feedback loops can accelerate cortical reorganization by reinforcing volitional attempts, even when overt movement is limited.

Furthermore, VR environments deliver structured, enriched sensory feedback while maintaining a high motivational appeal [78,79]. They can simulate functional tasks relevant to daily life and incorporate progressive difficulty adjustments in real-time, thereby promoting goal-directed motor learning. Combining VR, robotics, sensory enhancement, and BCI paradigms offers a multimodal approach that intensifies and personalizes BMT. This flexibility accommodates individual differences in stroke presentation, cognitive capacity, and recovery trajectory, ultimately advancing outcomes more effectively than conventional, unilateral protocols.

In interlimb coupling, these technologies can carefully tailor bilateral forces, match rhythmic patterns across limbs, or display real-time feedback on whether the affected and unaffected limbs work together in synergy. By making invisible neural synergies visible—or amplifying specific coupling patterns—multimodal platforms further bolster the neuroplastic processes underlying bilateral coordination and cross-limb gains [20,69].

Taken together, these interlinked neurophysiological processes—spanning cortical rebalancing, sensorimotor synchronization, spinal CPG activation, use-dependent plasticity, cerebellar modulation, cross-education, interlimb coupling, and technologically assisted feedback—provide a comprehensive rationale for Bilateral Movement Training’s efficacy in stroke rehabilitation. By engaging bilateral networks at multiple levels of the neuraxis, BMT fosters an environment of heightened plasticity and improved motor coordination, culminating in more robust and enduring functional recovery.

## 5. Limitations

The central part of research studying the effects of bilateral movement training has focused on interventions involving bilateral arm training in its various forms. There is an apparent lack of studies incorporated into this literature research involving bilateral leg, combined bilateral leg and arm (quadrupedal), bilateral rhythmic leg, and/or combined leg and arm interventions to draw a balanced conclusion on the diversity, efficiency, and effects between the different bilateral training methods.

## 6. Conclusions

The objective of this review was to shed light on the underlying neurophysiological principles of the bilateral and interlimb movement strategies that led to positive clinical post-stroke rehabilitation outcomes. The results show the critical role of bilateral movement training (BMT) and interlimb coupling in post-stroke rehabilitation, demonstrating their efficacy in enhancing motor function, promoting neuroplasticity, and improving overall recovery. BMT, particularly when integrated with robotic assistance, sensory enhancement, and virtual reality, offers a robust framework for maximizing rehabilitation outcomes.

A key strength of BMT lies in its ability to engage neurophysiological mechanisms such as central pattern generators, interhemispheric coupling, cortical disinhibition, and quadrupedal transfer, facilitating neural plasticity and interlimb coordination. High-intensity BMT is particularly beneficial for patients with moderate to severe motor impairments, while low-intensity training remains valuable for patients in the early stages of recovery.

Despite its promise, research gaps persist, particularly in the areas of long-term functional outcomes, patient stratification, and individualized rehabilitation protocols. The review highlights the importance of conducting longitudinal studies to evaluate the durability of motor improvements and the effects of advanced rehabilitation technologies. Additionally, psychosocial factors, including emotional well-being and motivation, must be integrated into rehabilitation strategies to enhance patient engagement.

To optimize recovery, future research should focus on personalized rehabilitation approaches, combined upper and lower limb training, and the neurophysiological mechanisms underlying the benefits of BMT. Integrating robotic assistance, virtual reality, and quadrupedal training holds promise for advancing stroke rehabilitation. By addressing these gaps, rehabilitation programs can become more patient-centered, evidence-based, and practical, ultimately improving stroke survivors’ functional independence and quality of life.

## 7. Recommendations and Future Directions

The findings in this paper advocate for a multidisciplinary approach incorporating advanced technologies and innovative methods to optimize recovery outcomes. Future research should focus on refining these strategies, particularly in understanding the specific protocols, such as interlimb coupling mechanisms and translation into de novo quadrupedal training protocols, and identifying conditions that yield the most effective results to enhance the quality of care for stroke survivors.

## Figures and Tables

**Table 1:** Specific definitions of terms used throughout this paper.

	Definition	Relevance	Authors/Source
**Bilateral Movement Training (BMT)**	Bilateral movement training in post-stroke rehabilitation involves the simultaneous use of both limbs to perform tasks, promoting coordination and functional recovery.	This method leverages the concept of neural plasticity, facilitating the reorganization of the brain’s neural networks and motor control organization.	Cauraugh, J. H., & Summers, J. J. Neural plasticity and bilateral movements: A rehabilitation approach for chronic stroke. *Progress in Neurobiology*, 75(5), 309–320. [27]
**Interlimb Coupling**	Interlimb coupling in stroke rehabilitation refers to the coordination between the movements of both limbs, which can influence motor recovery and functional performance.	Interlimb coupling exercises aim to exploit neural mechanisms that link the movements of the limbs, thereby facilitating the recovery of motor function in the affected limb through synchronized bilateral activities.	Schaefer, S. Y., & Lang, C. E. Using dual tasks to test immediate transfer of training between naturalistic movements: a proof-of-principle study. *Journal of Motor Behavior*, 44(5), 313–318. [31]
**Interlimb Transfer**	Interlimb transfer in stroke rehabilitation refers to the phenomenon where training or practicing a motor skill with one limb improves the performance of the same skill with the untrained contralateral limb.	This allows therapists to leverage the unaffected limb to enhance motor recovery in the affected limb(s).	Cauraugh, J. H., Kim, S. Two coupled motor recovery protocols are better than one: Electromyogram-triggered neuromuscular stimulation and bilateral movements. *Stroke*, 33(6). [32]
**Cross Education**	Cross-education in post-stroke rehabilitation refers to the phenomenon where strength training of one limb can lead to strength gains in the contralateral, untrained limb.	This effect is particularly beneficial in stroke rehabilitation, as exercising the unaffected limb can help improve strength and function in the affected limb, aiding overall recovery.	Farthing, J. P., & Zehr, E. P. Restoring symmetry: Clinical applications of cross-education. *Exercise and Sport Sciences Reviews*, 42(2), 70–75. [5]
**Bilateral Synergy**	Bilateral synergy in post-stroke rehabilitation refers to the coordinated and simultaneous use of both limbs to enhance motor recovery and functional performance.	This concept leverages the interconnectedness of the hemispheres in the brain, encouraging the non-affected limb to assist in rehabilitating the affected limb, thereby improving overall motor function and reducing asymmetry in movement patterns.	Lewis, G. N., & Perreault, E. J. The side of stroke affects interlimb coordination during passive movement. *Neurorehabilitation and Neural Repair*, 21(4), 280–285. [33]
**Interlimb Connections**	Interlimb connections in post-stroke rehabilitation refer to the neural pathways and mechanisms that facilitate communication and coordination between the limbs.	Interlimb connections are crucial for motor recovery. They enable the unaffected limb to support the rehabilitation of the affected limb by promoting symmetrical movement patterns and improving overall motor function and recovery.	Cauraugh, J. H., & Summers, J. J. Neural plasticity and bilateral movements: A rehabilitation approach for chronic stroke. *Progress in Neurobiology*, 75(5), 309–320. [27]
**Central Pattern Generators (CPG)**	Central pattern generators (CPGs) in stroke rehabilitation refer to neural networks in the spinal cord that can produce rhythmic patterned outputs, such as walking or other repetitive movements, without sensory feedback.	These neural circuits facilitate motor recovery by enabling rhythmic and coordinated movement patterns. Therapeutic interventions can harness and retrain these patterns to improve functional mobility in stroke patients.	Dietz, V. Spinal cord pattern generators for locomotion. *Clinical Neurophysiology*, 114(8), 1379–1389. [23]

**Table 2:** Effects of bilateral movement training, interlimb coupling in post-stroke rehabilitation, and potential neurophysiological mechanisms underpinning intervention effect.

Intervention Type and Authors	Participants (Sex/Number/Age)	Measurement(s)	Effect on Stroke Condition	Neurophysiological, Interlimb Coupling, and Transfer Effects *	No. of Potential Facilitating Neurophysiological Mech.
**I. BILATERAL ARM TRAINING**					
**Bruyneel, et al. [57]**	n/a-15 poststroke 17 healthy volunteers-n/a	CMSA/Levin Scale/Ashworth/Semmes–Weinstein/Box and Blocks	Bilateral pushing with gradual efforts induces impaired postural strategies and coordination between limbs in individuals after a stroke.	1, 2, 3, 4, 5, 7, and 8	7
**Dhakate, D., & Bhattad, R. [58]**	n/a-40 post-stroke subjects-45–65	FIM (Functional Independence Measure) and FMA UE (Fugl-Meyer et al.)	Bilateral arm training proved more effective than the Conventional Training program in improving affected upper extremity motor function.	1, 2, 3, 4, 5, 6, and 8	7
**Duff, et al. [59]**	M/F, 20 post-stroke/20 healthy controls	Adult Assisting Hand Assessment (Ad-AHA Stroke) and UE Fugl-Meyer (UEFM)	Algorithm and sensor data analyses distinguished task types within and between groups and predicted clinical scores.	1, 2, 3, 4, 5, 6, 7, and 8	8
**Han, K. J., & Kim, J. Y. [29]**	n/a, 30 post-stroke subjects, n/a	FMA UE/ Box and Blocks/ MBI (Modified Barthel Index	In both the experimental and control groups, the FMA, BBT, and MBI scores were significantly higher after the intervention than before the intervention (*p* < 0.05). The changes in the FMA, BBT, and MBI scores were more significant in the experimental group than in the control group (*p* < 0.05).	1, 2, 3, 4, 5, 6, and 8	7
**Itkonen, M., et al. [60]**	M/F, 11 post-stroke subjects, 52–90	Surface EMG measurements	The paretic arms of the patients were more strongly affected by the task conditions compared with the non-paretic arms. These results suggest that in-phase motion may activate neural circuits that trigger recovery.	1, 2, 3, 4, 5, 7, and 8	7
**Kim, N., et al. [61]**	n/a, 13 hemiparetic stroke patients and 12 healthy participants, n/a	EMG data	The upper extremity muscle activities of stroke patients during bimanual tasks varied between the paretic and non-paretic sides. Interestingly, the non-paretic side muscle activities also differed from regular participants.	1, 2, 3, 4, 5, 6, and 8	7
**Kumagai, M., et al. [62]**	M/F, 24 subjects, n/a	NHPT, Purdue Pegboard task, Box and Blocks test, FMA UE	Alternating bilateral training may augment training effects and improve upper limb motor function in patients with left hemiparesis.	1, 2, 3, 4, 5, 6, and 8	7
**Lee, M. J., et al. [38]**	M/F, 15 post-stroke, 15 healthy, n/a	FMA UE, Box and Blocks test, MBI	Bilateral arm training and general occupational therapy might be more effective together than alone for improving upper limb function and ADL performance.	1, 2, 3, 4, 5, 6, and 8	7
**Meng, G., et al. [63]**	M/F, 128 subjects	FMA UE and Action research Reach Test Secondary: Neurophysiological improvement TMS	Hand–arm intensive bilateral training significantly improved motor functional and neurophysiological outcomes in patients with acute stroke.	1, 2, 3, 4, 5, 6, and 8	7
**Kaupp, C., et al. [24]**	M/F, 19 subjects, 57–87 y/o	MAS, Chedoke, Monofilaments sensory discrimination, Berg Balance Test	Results show significant changes in function and neurophysiological integrity.	1, 2, 3, 4, 5, 8, 13, 14, and 15	9
**II. BILATERAL ARM TRAINING AND SENSORY ENHANCEMENT**					
**Lin, C.H, et al. [64]**	M/F, 33 subjects, mean age = 55.1 ± 10.5,	BI, FMA UE, WMFT, MAS	Computer-aided interlimb force coupling training improves the motor recovery of a paretic hand. It facilitates motor control and enhances functional performance in the paretic upper extremity of people with chronic stroke.	1, 2, 3, 4, 5, 6, 7, 8, 9, and 11	9
**Rodrigues, L. C., et al. [65]**	M/F, 26 subjects, n/a	The primary outcome measure was unilateral and bilateral UL activity according to the Test d’Évaluation des Membres Supérieurs de Personnes Âgées (TEMPA).	The total TEMPA score showed the main effect of time. Significant improvement was found for bilateral but not unilateral tasks. Both groups showed gains after training, with no differences between them.	1, 2, 3, 4, 5, 7, and 8	7
**Song, G. B. [66].**	M/F, 40 subjects, mean age 51.15 ± 14.81 years,	Box and Block test (BBT), Jebsen Taylor test (JBT), and Modified Barthel Index (MBI)	Upper limb function and the ability to perform activities of daily living improved significantly in both groups. Although there were significant differences between the groups, the task-oriented group showed more remarkable improvement in upper limb function and activities of daily living.	1, 2, 3, 4, 5, 6, 7, 8, 9, and 17	10
**Van Delden, A. L. E. Q, et al. [67]**	M/F, 60 subjects, n/a	Potentiometer, smoothness, and harmony mean amplitude and bimanual coordination measurements.	The coupling between both hands was not significantly higher after bilateral than unilateral training and control treatment. BATRAC group showed greater movement harmonicity and larger amplitudes.	1, 2, 3, 4, 5, 7, 8, 9, and 17	9
**III. BILATERAL ARM TRAINING AND ROBOTICS**					
**Abdollahi, F., et al. [68]**	M/F, 26 subjects, 26–77 y/o	FMA/ Wolf Motor Functional Ability Scale (WMFAS)/Motor activity log	Subjects’ 2-week gains in Fugl-Meyer score averaged 2.92, and we also observed improvements in Wolf Motor Functional Ability Scale average of 0.21 and Motor Activity Log of 0.58 for quantity and 0.63 for quality of life scores.	1, 2, 3, 4, 5, 8, 9, 10, 11, and 16	10
**Huang, J. J., et al. [69]**	n/a, 40 subjects, n/a	EEG measurements	The results showed that stroke duration might influence the effects of hand rehabilitation in bilateral cortical corticocortical communication with significant main effects under different alpha and beta band conditions.	1, 2, 3, 4, 5, 6, 8, 9, and 10	9
**Li, Y. C., et al. [13]**	F/M, 72 subjects, 20 to 80 y/o	FMA UE/MAS/ABIL hand stroke impact scale/lateral pinch/accelerometer	Only between-group differences were detected for the primary outcome, FMA-UE. R-mirr enhanced upper limb motor improvement more effectively, and the effect could be maintained at 3 months of follow-up.	1, 2, 3, 4, 5, 6, 8, 9, and 16	9
**IV. BAT AND VIRTUAL REALITY/VIDEO GUIDANCE**					
**Jayasinghe, S. A., et al. [70]**	M/F, 15 stroke survivors and seven age-matched neurologically intact adults, 45–79 y/o	Fugl-Meyer, Jebsen Taylor	Chronic stroke survivors with mild hemiparesis show significant deficits in reaching aspects of bilateral coordination. However, there are no deficits in stabilizing against a movement-dependent spring load.	1, 2, 3, 4, 5, 8	6
**V. BILATERAL LEG TRAINING**					
**Ardestani, et al. [71]**	M/F, 50 subjects, 18–85 y/o	FMA UE, Changes in spatiotemporal, joint kinematics, and kinetics plus heart physiology variables were measured	High-intensity LT results in greater changes in kinematics and kinetics than lower-intensity interventions. The results may suggest greater paretic limb contributions.	1, 2, 3, 4, 5, 6, 8, 12, 13, and 15	10
**Jo, P. Y. [72]**	M/F, 20 subjects, n/a	The primary clinical measure was a 10 m walk time. Additional measures were the Timed test and the Stroke Impact Scale 3.0	Interlimb symmetry and knee–ankle variability post-stroke relate to walking performance. Interlimb angle–angle asymmetry does not relate to walking performance post-stroke.	1, 2, 3, 4, 5, 6, 8, 12, 13, and 15	10
**VI. BILATERAL LEG TRAINING PLUS SENSORY ENHANCEMENT**					
**Kwong, P.W.H., et al. [73]**	M/F, 72 subjects, 55–85 y/0	The muscle strength of paretic ankle dorsiflexors (pDFs), plantarflexors (pPFs), paretic knee extensors (pKEs), flexors (pKFs) were selected as the primary outcome measures of this study.	The application of bilateral TENS over the common peroneal nerve combined with TOT was superior to that of unilateral TENS combined with TOT in improving paretic ankle dorsiflexion strength.	1, 2, 3, 4, 5, 6, 7, 8, 9, and 12	10
**VII. COMBINED BILATERAL ARM AND LEG TRAINING**					
**Arya et al. [15]**	M/F, 50 subjects, n/a	The outcome measures included the feasibility of activities, Fugl-Meyer assessment (FMA), Rivermead Visual Gait Assessment (RVGA), Functional Ambulation Category (FAC), and modified Rankin Scale (mRS).	Interlimb coupling training, a feasible program, may enhance stroke recovery in both the upper and lower limbs, as well as gait.	1, 2, 3, 4, 5, 6, 7, 8, 12, 13, 14, 15, and 16	13
**VIII. BILATERAL RHYTHMIC LEG AND ARM TRAINING**					
**Klarner, T., et al. [25]**	M/F, 19 subjects, 45–86 y/o	Test for muscle tone (modified Ashworth), functional ambulation (FAC), physical impairment (Chedoke–McMaster scale), touch discrimination (monofilament test), and reflex function for stroke participants.	Arm and leg cycling training induces plasticity and modifies reflex excitability after stroke.	1, 2, 3, 4, 5, 8, 12, 13, 14, and 15	10
**IX. BILATERAL MOVEMENT PRIMING**					
**Stoykov, M. E., et al. [74]**	F/M, 76 subjects,	The primary outcome measure is the Fugl-Meyer Test of Upper Extremity Function. The secondary outcome is the Chedoke Arm and Hand Activity Index-Nine, an assessment of bimanual functional tasks.	The first large-scale clinical trial of bilateral priming plus task-specific training. The authors have previously conducted a feasibility study on bilateral motor priming plus task-specific training and have considerable experience using this protocol. Outcome follows.	1, 2, 3, 4, 5, 8, 9, 13, 15, and 16	10

* 1. Engaging both hemispheres and reducing inhibition in the affected cerebral cortex, leveraging interhemispheric coupling and neural cross-talk [75]. 2. Bilateral arm training induces more trunk muscle contractions, leading to better control of the proximal upper extremity and facilitating the expression of brain-derived neurotrophic factors and brain function remodeling [75]. 3. Facilitating neuroplasticity [75]. 4. Intact neural circuits within the spinal cord remain relatively unimpaired and accessible [75]. 5. The maintenance of spatial and temporal coupling after stroke is often (partially) intact and can be used in stroke rehabilitation [76]. 6. Applying meaningful, motivated tasks [76]. 7. In-phase motion of bilateral training causes more muscle synergy, especially in the affected arm [60]. 8. Transferability of skills acquired through bilateral training to unilateral tasks [54]. 9. Sensory enhancement can amplify interlimb reflexes and enhance motor learning and coordination. Sensory enhancement can modulate functional connectivity in sensory–motor networks and improve sensorimotor adaptation [77]. 10. Passive robot-controlled arm movements and proprioceptive decision-making and feedback have been shown to modulate functional connectivity in sensory–motor networks and enhance sensorimotor adaptation [77]. 11. Adding virtual reality and video guidance targets motor function and stimulates cognitive and perceptual processes, providing a more comprehensive approach to rehabilitation [78,79]. 12. Bilateral leg movement training has been associated with increased activation of the non-affected motor cortex during paretic leg movements, indicating neuroplastic changes [45]. 13. Antiphase oscillatory effects on central pattern generators (CPGs) [15]. Central pattern-generating networks (CPGs) are believed to be central to spinal circuits, assisting in producing rhythmic coordinated movements of all four limbs [23]. 14. Quadrupedic interlimb transfer with arm training: rhythmic movements of the arms impact reflexes in the lower limbs, resulting in both inhibitory [80,81] and facilitative effects [82]. 15. Interlimb coupling effects can facilitate bilateral motor output during rhythmic leg cycling after stroke [12]. Active rhythmic arm movements have been found to modulate the corticospinal drive to the legs, suggesting a potential mechanism for enhancing bilateral motor function [83]. 16. Bilateral movement priming increases corticomotor excitability in the primary motor cortex [84] and improves motor learning and recovery [85]. 17. Rhythmic auditory cues can significantly improve gait parameters and motor performance in individuals with neurological conditions like stroke and Parkinson’s disease [86].

## Data Availability

All data are included in the publication.

## Supplementary Materials

The following supporting information can be downloaded at https://www.mdpi.com/article/10.3390/jcm14113757/s1. File S1: PRISMA 2020 Checklist; File S2: Risk of BIAS assessment of the included research papers.

## References

[1] Stoykov M.E., Madhavan S. (2015). Motor priming in neurorehabilitation. J. Neurol. Phys. Ther..

[2] Stewart K.C., Cauraugh J.H., Summers J.J. (2006). Bilateral movement training and stroke rehabilitation: A systematic review and meta-analysis. J. Neurol. Sci..

[3] Stinear C.M., Byblow W.D., Ward S.H. (2014). An update on predicting motor recovery after stroke. Ann. Phys. Rehabil. Med..

[4] Kwakkel G., Veerbeek J.M., Van Wegen E.E.H., Wolf S.L. (2015). Constraint-induced movement therapy after stroke. Lancet Neurol..

[5] Farthing J.P., Zehr E.P. (2014). Restoring symmetry: Clinical applications of cross-education. Exerc. Sport Sci. Rev..

[6] Lee M., Carroll T.J. (2007). Cross education: Possible mechanisms for the contralateral effects of unilateral resistance training. Sports Med..

[7] Harjpal P., Qureshi M.d.I., Kovela R.K., Jain M. (2022). Bilateral lower limb training for post-stroke survivors: A bibliometric analysis. Cureus.

[8] Cauraugh J.H., Lodha N., Naik S.K., Summers J.J. (2010). Bilateral movement training and stroke motor recovery progress: A structured review and meta-analysis. Hum. Mov. Sci..

[9] Liu Y., Tang Y., Wang L., Yu P., Wang C., Zeng L., Yuan J., Zhao L. (2024). Optimal acupuncture methods for lower limb motor dysfunction after stroke: A systematic review and network meta-analysis. Front. Neurol..

[10] Timmermans A.A., Seelen H.A., Willmann R.D., Kingma H. (2009). Technology-assisted training of arm-hand skills in stroke: Concepts on reacquisition of motor control and therapist guidelines for rehabilitation technology design. J. Neuroeng. Rehabil..

[11] Vasudevan E.V., Kirk E.M., Jensen W., Andersen O.K., Akay M. (2014). Improving interlimb coordination following stroke: How can we change how people walk (and why should we)?. Replace, Repair, Restore, Relieve—Bridging Clinical and Engineering Solutions in Neurorehabilitation.

[12] Zehr E.P., Barss T.S., Kaupp C., Klarner T., Mezzarane R.A., Nakajima T., Sun Y., Komiyama T., Jensen W., Andersen O.K., Akay M. (2014). Neuromechanical interlimb interactions and rehabilitation of walking after stroke. Replace, Repair, Restore, Relieve—Bridging Clinical and Engineering Solutions in Neurorehabilitation.

[13] Li K.-P., Wu J.-J., Zhou Z.-L., Xu D.-S., Zheng M.-X., Hua X.-Y., Xu J.-G. (2023). Noninvasive brain stimulation for neurorehabilitation in post-stroke patients. Brain Sci..

[14] Maceira-Elvira P., Popa T., Schmid A.-C., Hummel F.C. (2019). Wearable technology in stroke rehabilitation: Towards improved diagnosis and treatment of upper-limb motor impairment. J. Neuroeng. Rehabil..

[15] Arya K.N., Pandian S., Sharma A., Kumar V., Kashyap V.K. (2020). Interlimb coupling in poststroke rehabilitation: A pilot randomized controlled trial. Top. Stroke Rehabil..

[16] Khan A., Malik A., Yaseen A., Mahmood T., Nazir M., Khan S. (2023). Mobility related confidence level in chronic stroke patients through task oriented walking intervention. Super. J. Phys. Ther. Rehabil..

[17] Keeling A.B., Piitz M., Semrau J.A., Hill M.D., Scott S.H., Dukelow S.P. (2021). Robot enhanced stroke therapy optimizes rehabilitation (restore): A pilot study. J. Neuroeng. Rehabil..

[18] Hesse S., Mehrholz J., Werner C. (2008). Robot-assisted upper and lower limb rehabilitation after stroke: Walking and arm/hand function. Dtsch. Arztebl. Int..

[19] Cauraugh J.H., Kang N. (2021). Bimanual movements and chronic stroke rehabilitation: Looking back and looking forward. Appl. Sci..

[20] Hatem S.M., Saussez G., Della Faille M., Prist V., Zhang X., Dispa D., Bleyenheuft Y. (2016). Rehabilitation of motor function after stroke: A multiple systematic review focused on techniques to stimulate upper extremity recovery. Front. Hum. Neurosci..

[21] French B., Thomas L.H., Coupe J., McMahon N.E., Connell L., Harrison J., Sutton C.J., Tishkovskaya S., Watkins C.L. (2016). Repetitive task training for improving functional ability after stroke. Cochrane Database Syst. Rev..

[22] Yoon J.A., Koo B.I., Shin M.J., Shin Y.B., Ko H.-Y., Shin Y.-I. (2014). Effect of constraint-induced movement therapy and mirror therapy for patients with subacute stroke. Ann. Rehabil. Med..

[23] Dietz V. (2003). Spinal cord pattern generators for locomotion. Clin. Neurophysiol..

[24] Kaupp C., Pearcey G.E.P., Klarner T., Sun Y., Cullen H., Barss T.S., Zehr E.P. (2018). Rhythmic arm cycling training improves walking and neurophysiological integrity in chronic stroke: The arms can give legs a helping hand in rehabilitation. J. Neurophysiol..

[25] Klarner T., Barss T., Sun Y., Kaupp C., Loadman P., Zehr E. (2016). Long-term plasticity in reflex excitability induced by five weeks of arm and leg cycling training after stroke. Brain Sci..

[26] Whitall J., Waller S.M., Silver K.H.C., Macko R.F. (2000). Repetitive bilateral arm training with rhythmic auditory cueing improves motor function in chronic hemiparetic stroke. Stroke.

[27] Cauraugh J.H., Summers J.J. (2005). Neural plasticity and bilateral movements: A rehabilitation approach for chronic stroke. Prog. Neurobiol..

[28] Summers J.J., Kagerer F.A., Garry M.I., Hiraga C.Y., Loftus A., Cauraugh J.H. (2007). Bilateral and unilateral movement training on upper limb function in chronic stroke patients: A tms study. J. Neurol. Sci..

[29] Han K.J., Kim J.Y. (2016). The effects of bilateral movement training on upper limb function in chronic stroke patients. J. Phys. Ther. Sci..

[30] Harjpal P., Qureshi M.d.I., Kovela R.K., Jain M. (2021). Efficacy of bilateral lower limb training over unilateral to re-educate balance and walking in post-stroke survivors: A protocol for randomized clinical trial. J. Pharm. Res. Int..

[31] Schaefer S.Y., Lang C.E. (2012). Using dual tasks to test immediate transfer of training between naturalistic movements: A proof-of-principle study. J. Mot. Behav..

[32] Cauraugh J.H., Kim S. (2002). Two coupled motor recovery protocols are better than one: Electromyogram-triggered neuromuscular stimulation and bilateral movements. Stroke.

[33] Lewis G.N., Perreault E.J. (2007). Side of lesion influences bilateral activation in chronic, post-stroke hemiparesis. Clin. Neurophysiol..

[34] Schick T., Kolm D., Leitner A., Schober S., Steinmetz M., Fheodoroff K. (2022). Efficacy of four-channel functional electrical stimulation on moderate arm paresis in subacute stroke patients—Results from a randomized controlled trial. Healthcare.

[35] Bolton D.A.E., Cauraugh J.H., Hausenblas H.A. (2004). Electromyogram-triggered neuromuscular stimulation and stroke motor recovery of arm/hand functions: A meta-analysis. J. Neurol. Sci..

[36] Dietz V., Holliger N.S., Christen A., Geissmann M., Filli L. (2024). Neural coordination of bilateral hand movements: Evidence for an involvement of brainstem motor centres. J. Physiol..

[37] Allen J.L., Ting L.H., Kesar T.M. (2018). Gait rehabilitation using functional electrical stimulation induces changes in ankle muscle coordination in stroke survivors: A preliminary study. Front. Neurol..

[38] Lee D.H., Park S.H., Han J.W. (2017). Effect of bilateral upper extremity exercise on trunk performance in patients with stroke. J. Phys. Ther. Sci..

[39] Richardson M.C., Tears C., Morris A., Alexanders J. (2021). The effects of unilateral versus bilateral motor training on upper limb function in adults with chronic stroke: A systematic review. J. Stroke Cerebrovasc. Dis..

[40] Chen Y.-W., Lin K.-c., Li Y.-c., Lin C.-J. (2023). Predicting patient-reported outcome of activities of daily living in stroke rehabilitation: A machine learning study. J. Neuroeng. Rehabil..

[41] Renner C.I.E., Brendel C., Hummelsheim H. (2020). Bilateral arm training vs unilateral arm training for severely affected patients with stroke: Exploratory single-blinded randomized controlled trial. Arch. Phys. Med. Rehabil..

[42] Chen S., Qiu Y., Bassile C.C., Lee A., Chen R., Xu D. (2022). Effectiveness and success factors of bilateral arm training after stroke: A systematic review and meta-analysis. Front. Aging Neurosci..

[43] Dembele J., Triccas L.T., Amanzonwé L.E.R., Kossi O., Spooren A. (2024). Bilateral versus unilateral upper limb training in (sub)acute stroke: A systematic and meta-analysis. S. Afr. J. Physiother..

[44] Sethy D., Bajpai P., Kujur E.S., Mohakud K., Sahoo S. (2016). Effectiveness of modified constraint induced movement therapy and bilateral arm training on upper extremity function after chronic stroke: A comparative study. Open J. Ther. Rehabil..

[45] Crosby L.D., Marrocco S., Brown J., Patterson K.K. (2016). A novel bilateral lower extremity mirror therapy intervention for individuals with stroke. Heliyon.

[46] Chang W.H., Kim Y.-H. (2013). Robot-assisted therapy in stroke rehabilitation. J. Stroke.

[47] Shiner C.T., Byblow W.D., McNulty P.A. (2014). Bilateral priming before wii-based movement therapy enhances upper limb rehabilitation and its retention after stroke: A case-controlled study. Neurorehabil. Neural Repair.

[48] Chen P.-m., Kwong P.W.H., Lai C.K.Y., Ng S.S.M. (2019). Comparison of bilateral and unilateral upper limb training in people with stroke: A systematic review and meta-analysis. PLoS ONE.

[49] Stinear C.M., Petoe M.A., Anwar S., Barber P.A., Byblow W.D. (2014). Bilateral priming accelerates recovery of upper limb function after stroke: A randomized controlled trial. Stroke.

[50] Waller M.S., Whitall J., Jenkins T., Magder L.S., Hanley D.F., Goldberg A., Luft A.R. (2014). Sequencing bilateral and unilateral task-oriented training versus task oriented training alone to improve arm function in individuals with chronic stroke. BMC Neurol..

[51] Van Delden A.L.E.Q., Peper C.L.E., Kwakkel G., Beek P.J. (2012). A systematic review of bilateral upper limb training devices for poststroke rehabilitation. Stroke Res. Treat..

[52] Syed N., Biswas A., Hanifa N., Rv P., Sundaram P. (2015). Bilateral versus unilateral upper extremity training on upper limb motor activity in hemiplegia. Int. J. Neurorehabilit..

[53] Shih P.-C., Steele C.J., Hoepfel D., Muffel T., Villringer A., Sehm B. (2023). The impact of lesion side on bilateral upper limb coordination after stroke. J. Neuroeng. Rehabil..

[54] Wang J., Lei Y., Xiong K., Marek K. (2013). Substantial generalization of sensorimotor learning from bilateral to unilateral movement conditions. PLoS ONE.

[55] Wu C.-y., Chuang L.-l., Lin K.-c., Chen H.-c., Tsay P.-k. (2011). Randomized trial of distributed constraint-induced therapy versus bilateral arm training for the rehabilitation of upper-limb motor control and function after stroke. Neurorehabil. Neural Repair.

[56] Langan J., Doyle S.T., Hurvitz E.A., Brown S.H. (2010). Influence of task on interlimb coordination in adults with cerebral palsy. Arch. Phys. Med. Rehabil..

[57] Bruyneel A.-V., Higgins J., Akremi H., Aissaoui R., Nadeau S. (2021). Postural organization and inter-limb coordination are altered after stroke when an isometric maximum bilateral pushing effort of the upper limbs is performed. Clin. Biomech..

[58] Dhakate D., Bhattad R. (2020). Study the effectiveness of bilateral arm training on upper extremity motor function and activity level in patients with sub-acute stroke. Int. J. Curr. Res. Rev..

[59] Duff S.V., Miller A., Quinn L., Youdan G., Bishop L., Ruthrauff H., Wade E. (2022). Quantifying intra- and interlimb use during unimanual and bimanual tasks in persons with hemiparesis post-stroke. J. Neuroeng. Rehabil..

[60] Itkonen M., Costa Á., Yamasaki H., Okajima S., Alnajjar F., Kumada T., Shimoda S. (2019). Influence of bimanual exercise on muscle activation in post-stroke patients. ROBOMECH J..

[61] Kim N., Jo S., Bae K., Song C. (2022). Comparison of upper extremity muscle activity between stroke patients and healthy participants while performing bimanual tasks. Phys. Ther. Rehabil. Sci..

[62] Kumagai M., Uehara S., Kurayama T., Kitamura S., Sakata S., Kondo K., Shimizu E., Yoshinaga N., Otaka Y. (2022). Effects of alternating bilateral training between non-paretic and paretic upper limbs in patients with hemiparetic stroke: A pilot randomized controlled trial. J. Rehabil. Med..

[63] Meng G., Meng X., Tan Y., Yu J., Jin A., Zhao Y., Liu X. (2018). Short-term efficacy of hand-arm bimanual intensive training on upper arm function in acute stroke patients: A randomized controlled trial. Front. Neurol..

[64] Lin C.-H., Chou L.-W., Luo H.-J., Tsai P.-Y., Lieu F.-K., Chiang S.-L., Sung W.-H. (2015). Effects of computer-aided interlimb force coupling training on paretic hand and arm motor control following chronic stroke: A randomized controlled trial. PLoS ONE.

[65] Rodrigues L.C., Farias N.C., Gomes R.P., Michaelsen S.M. (2016). Feasibility and effectiveness of adding object-related bilateral symmetrical training to mirror therapy in chronic stroke: A randomized controlled pilot study. Physiother. Theory Pract..

[66] Song G.B. (2015). The effects of task-oriented versus repetitive bilateral arm training on upper limb function and activities of daily living in stroke patients. J. Phys. Ther. Sci..

[67] Van Delden A.L.E.Q., Beek P.J., Roerdink M., Kwakkel G., Peper C.L.E. (2015). Unilateral and bilateral upper-limb training interventions after stroke have similar effects on bimanual coupling strength. Neurorehabil. Neural Repair.

[68] Abdollahi F., Corrigan M., Lazzaro E.D.C., Kenyon R.V., Patton J.L. (2018). Error-augmented bimanual therapy for stroke survivors. NeuroRehabilitation.

[69] Huang J.-J., Pei Y.-C., Chen Y.-Y., Tseng S.-S., Hung J.-W. (2022). Bilateral sensorimotor cortical communication modulated by multiple hand training in stroke participants: A single training session pilot study. Bioengineering.

[70] Jayasinghe S.A.L., Maenza C., Good D.C., Sainburg R.L. (2021). Deficits in performance on a mechanically coupled asymmetrical bilateral task in chronic stroke survivors with mild unilateral paresis. Symmetry.

[71] Ardestani M.M., Henderson C.E., Mahtani G., Connolly M., Hornby T.G. (2020). Locomotor kinematics and kinetics following high-intensity stepping training in variable contexts poststroke. Neurorehabil. Neural Repair.

[72] Jo P.Y. (2019). Intralimb Coordination and Intermuscular Coherence in Walking After Stroke. Ph.D. Thesis.

[73] Kwong P.W.H., Ng G.Y.F., Chung R.C.K., Ng S.S.M. (2018). Bilateral transcutaneous electrical nerve stimulation improves lower-limb motor function in subjects with chronic stroke: A randomized controlled trial. J. Am. Heart Assoc..

[74] Stoykov M.E., King E., David F.J., Vatinno A., Fogg L., Corcos D.M. (2020). Bilateral motor priming for post stroke upper extremity hemiparesis: A randomized pilot study. Restor. Neurol. Neurosci..

[75] Kiper P., Godart N., Cavalier M., Berard C., Cieślik B., Federico S., Kiper A., Pellicciari L., Meroni R. (2023). Effects of immersive virtual reality on upper-extremity stroke rehabilitation: A systematic review with meta-analysis. J. Clin. Med..

[76] Cazac G.I. (2022). Virtual reality based-therapy: Targeting balance impairments to improve gait in stroke. Știința și Arta Mișcării.

[77] Sun Y., Zehr E.P. (2019). Sensory enhancement amplifies interlimb cutaneous reflexes in wrist extensor muscles. J. Neurophysiol..

[78] Lee S.-H., Kim Y.-M., Lee B.-H. (2015). Effects of virtual reality-based bilateral upper-extremity training on brain activity in post-stroke patients. J. Phys. Ther. Sci..

[79] Song Y.-H., Lee H.-M. (2021). Effect of immersive virtual reality-based bilateral arm training in patients with chronic stroke. Brain Sci..

[80] Frigon A., Collins D.F., Zehr E.P. (2004). Effect of rhythmic arm movement on reflexes in the legs: Modulation of soleus h-reflexes and somatosensory conditioning. J. Neurophysiol..

[81] Zehr E.P., Balter J.E., Ferris D.P., Hundza S.R., Loadman P.M., Stoloff R.H. (2007). Neural regulation of rhythmic arm and leg movement is conserved across human locomotor tasks. J. Physiol..

[82] Dragert K., Zehr E.P. (2009). Rhythmic arm cycling modulates hoffmann reflex excitability differentially in the ankle flexor and extensor muscles. Neurosci. Lett..

[83] Zhou R., Alvarado L., Kim S., Chong S.L., Mushahwar V.K. (2017). Modulation of corticospinal input to the legs by arm and leg cycling in people with incomplete spinal cord injury. J. Neurophysiol..

[84] Jordan H.T., Stinear C.M. (2018). Effects of bilateral priming on motor cortex function in healthy adults. J. Neurophysiol..

[85] Madhavan S., Stinear J.W., Kanekar N. (2016). Effects of a single session of high intensity interval treadmill training on corticomotor excitability following stroke: Implications for therapy. Neural Plast..

[86] Ghai S., Ghai I. (2018). Effects of rhythmic auditory cueing in gait rehabilitation for multiple sclerosis: A mini systematic review and meta-analysis. Front. Neurol..

[87] Stoykov M.E., Corcos D.M. (2009). A review of bilateral training for upper extremity hemiparesis. Occup. Ther. Int..

[88] Stoykov M.E., Lewis G.N., Corcos D.M. (2009). Comparison of bilateral and unilateral training for upper extremity hemiparesis in stroke. Neurorehabil. Neural Repair.

[89] Lee M.-J., Lee J.-H., Koo H.-M., Lee S.-M. (2017). Effectiveness of bilateral arm training for improving extremity function and activities of daily living performance in hemiplegic patients. J. Stroke Cerebrovasc. Dis..

[90] Moran J., Ramirez-Campillo R., Liew B., Chaabene H., Behm D.G., García-Hermoso A., Izquierdo M., Granacher U. (2021). Effects of bilateral and unilateral resistance training on horizontally orientated movement performance: A systematic review and meta-analysis. Sports Med..

[91] Lin J., He J., Shu B., Jia J. (2020). Multi-sensory feedback therapy combined with task-oriented training on the hemiparetic upper limb in chronic stroke: Study protocol for a pilot randomized controlled trial. Res. Sq..

[92] Bayona N.A., Bitensky J., Salter K., Teasell R. (2005). The role of task-specific training in rehabilitation therapies. Top. Stroke Rehabil..

[93] Carlsson H., Rosén B., Björkman A., Pessah-Rasmussen H., Brogårdh C. (2021). Sensory re-learning of the upper limb (sensupp) after stroke: Development and description of a novel intervention using the tidier checklist. Trials.

[94] Amki M., Baumgartner P., Bracko O., Luft A.R., Wegener S. (2017). Task-specific motor rehabilitation therapy after stroke improves performance in a different motor task: Translational evidence. Transl. Stroke Res..

[95] Birkenmeier R.L., Prager E.M., Lang C.E. (2010). Translating animal doses of task-specific training to people with chronic stroke in 1-hour therapy sessions: A proof-of-concept study. Neurorehabil. Neural Repair.

[96] Dolbow D.R., Gorgey A.S., Recio A.C., Stiens S.A., Curry A.C., Sadowsky C.L., Gater D.R., Martin R., McDonald J.W. (2015). Activity-based restorative therapies after spinal cord injury: Inter-institutional conceptions and perceptions. Aging Dis..

[97] Cunningham P., Turton A.J., Van Wijck F., Van Vliet P. (2016). Task-specific reach-to-grasp training after stroke: Development and description of a home-based intervention. Clin. Rehabil..

[98] Khallaf M.E. (2020). Effect of task-specific training on trunk control and balance in patients with subacute stroke. Neurol. Res. Int..

[99] Grefkes C., Fink G.R. (2020). Recovery from stroke: Current concepts and future perspectives. Neurol. Res. Pract..

[100] Demers M., Varghese R., Winstein C.J. (2021). Retrospective exploratory analysis of task-specific effects on brain activity after stroke. medRxiv.

[101] Iqbal S., Saeed A., Batool S., Waqqar S., Khattak H.G., Jabeen H. (2023). Effects of task-oriented balance training with sensory integration in post stroke patients. Rehabil. J..

[102] Chiaramonte R., Bonfiglio M., Leonforte P., Coltraro G., Guerrera C., Vecchio M. (2022). Proprioceptive and dual-task training: The key of stroke rehabilitation, a systematic review. J. Funct. Morphol. Kinesiol..

[103] Hermann D.M., Bassetti C.L. (2016). Role of sleep-disordered breathing and sleep-wake disturbances for stroke and stroke recovery. Neurology.

[104] Chuang L.-L., Chen Y.-L., Chen C.-C., Li Y.-C., Wong A.M.-K., Hsu A.-L., Chang Y.-J. (2017). Effect of emg-triggered neuromuscular electrical stimulation with bilateral arm training on hemiplegic shoulder pain and arm function after stroke: A randomized controlled trial. J. Neuroeng. Rehabil..

[105] Alotaibi N.D., Jahanshahi H., Yao Q., Mou J., Bekiros S. (2023). Identification and control of rehabilitation robots with unknown dynamics: A new probabilistic algorithm based on a finite-time estimator. Mathematics.

[106] Shin S.S., Pelled G. (2017). Novel neuromodulation techniques to assess interhemispheric communication in neural injury and neurodegenerative diseases. Front. Neural Circuits.

[107] Du Q., Luo J., Cheng Q., Wang Y., Guo S. (2022). Vibrotactile enhancement in hand rehabilitation has a reinforcing effect on sensorimotor brain activities. Front. Neurosci..

[108] Jia J. (2022). Exploration on neurobiological mechanisms of the central–peripheral–central closed-loop rehabilitation. Front. Cell. Neurosci..

[109] Chen X., Liu F., Yan Z., Cheng S., Liu X., Li H., Li Z. (2018). Therapeutic effects of sensory input training on motor function rehabilitation after stroke. Medicine.

[110] Bolognini N., Russo C., Edwards D.J. (2016). The sensory side of post-stroke motor rehabilitation. Restor. Neurol. Neurosci..

[111] Xu G., Gao X., Pan L., Chen S., Wang Q., Zhu B., Li J. (2018). Anxiety detection and training task adaptation in robot-assisted active stroke rehabilitation. Int. J. Adv. Robot. Syst..

[112] Liu G., Cai H., Leelayuwat N. (2022). Intervention effect of rehabilitation robotic bed under machine learning combined with intensive motor training on stroke patients with hemiplegia. Front. Neurorobot..

[113] Fiore S., Battaglino A., Sinatti P., Sánchez-Romero E.A., Ruiz-Rodriguez I., Manca M., Gargano S., Villafañe J.H. (2023). The effectiveness of robotic rehabilitation for the functional recovery of the upper limb in post-stroke patients: A systematic review. Retos.

[114] Khor K.X., Chin P.J.H., Hisyam A.R., Yeong C.F., Narayanan A.L.T., Su E.L.M. Development of cr2-haptic: A compact and portable rehabilitation robot for wrist and forearm training. Proceedings of the 2014 IEEE Conference on Biomedical Engineering and Sciences (IECBES).

[115] Su D., Hu Z., Wu J., Shang P., Luo Z. (2023). Review of adaptive control for stroke lower limb exoskeleton rehabilitation robot based on motion intention recognition. Front. Neurorobot..

[116] Park J.H., Park G., Kim H.Y., Lee J.-Y., Ham Y., Hwang D., Kwon S., Shin J.-H. (2020). A comparison of the effects and usability of two exoskeletal robots with and without robotic actuation for upper extremity rehabilitation among patients with stroke: A single-blinded randomised controlled pilot study. J. Neuroeng. Rehabil..

[117] Park J.H., Chung Y. (2016). The effects of providing visual feedback and auditory stimulation using a robotic device on balance and gait abilities in persons with stroke: A pilot study. Phys. Ther. Rehabil. Sci..

[118] Ren Y., Wu Y.-N., Yang C.-Y., Xu T., Harvey R.L., Zhang L.-Q. (2017). Developing a wearable ankle rehabilitation robotic device for in-bed acute stroke rehabilitation. IEEE Trans. Neural Syst. Rehabil. Eng..

[119] Li Y.-c., Lin K.-c., Chen C.-l., Yao G., Chang Y.-j., Lee Y.-y., Liu C.-t., Chen W.-S. (2023). Three ways to improve arm function in the chronic phase after stroke by robotic priming combined with mirror therapy, arm training, and movement-oriented therapy. Arch. Phys. Med. Rehabil..

[120] Lee Y.-c., Li Y.-c., Lin K.-c., Yao G., Chang Y.-j., Lee Y.-y., Liu C.-t., Hsu W.-l., Wu Y.-h., Chu H.-t. (2022). Effects of robotic priming of bilateral arm training, mirror therapy, and impairment-oriented training on sensorimotor and daily functions in patients with chronic stroke: Study protocol of a single-blind, randomized controlled trial. Trials.

[121] Li Y., Fan T., Qi Q., Wang J., Qiu H., Zhang L., Wu X., Ye J., Chen G., Long J. (2021). Efficacy of a novel exoskeletal robot for locomotor rehabilitation in stroke patients: A multi-center, non-inferiority, randomized controlled trial. Front. Aging Neurosci..

[122] Ji E.-K., Lee S.-H. (2016). Effects of virtual reality training with modified constraint-induced movement therapy on upper extremity function in acute stage stroke: A preliminary study. J. Phys. Ther. Sci..

[123] Yang D. (2024). Construction of sports music integration training and performance practice system based on virtual reality technology. J. Electr. Syst..

[124] Cho G.H., Hwangbo G., Shin H.S. (2014). The effects of virtual reality-based balance training on balance of the elderly. J. Phys. Ther. Sci..

[125] Hassan M. (2023). Impact of instant visual guidance via video technology on the correction of some offensive skills performance errors in basketball. Asian J. Sports Med..

[126] Langhorne P., Coupar F., Pollock A. (2009). Motor recovery after stroke: A systematic review. Lancet Neurol..

[127] Mehrholz J., Pohl M., Platz T., Kugler J., Elsner B. (2018). Electromechanical and robot-assisted arm training for improving activities of daily living, arm function, and arm muscle strength after stroke. Cochrane Database Syst. Rev..

[128] Wonsetler E.C., Bowden M.G. (2017). A systematic review of mechanisms of gait speed change post-stroke. Part 2: Exercise capacity, muscle activation, kinetics, and kinematics. Top. Stroke Rehabil..

[129] Wang C.-Y., Miyoshi S., Chen C.-H., Lee K.-C., Chang L.-C., Chung J.-H., Shi H.-Y. (2020). Walking ability and functional status after post-acute care for stroke rehabilitation in different age groups: A prospective study based on propensity score matching. Aging.

[130] French M.A., Moore M.F., Pohlig R., Reisman D. (2015). Self-efficacy mediates the relationship between balance/walking performance, activity, and participation after stroke. Top. Stroke Rehabil..

[131] Jeon H.J., Hwang B.Y. (2018). Effect of bilateral lower limb strengthening exercise on balance and walking in hemiparetic patients after stroke: A randomized controlled trial. J. Phys. Ther. Sci..

[132] Kalyana S., Lohar D., Khan J., Rao L.S. (2023). Effectiveness of supplemented backward walking training along with conventional therapy on balance and functional outcome in patients with stroke. Int. J. Curr. Pharm. Res..

[133] Cho K.H., Song W.-K. (2015). Robot-assisted reach training for improving upper extremity function of chronic stroke. Tohoku J. Exp. Med..

[134] Louie D.R., Mortenson W.B., Durocher M., Schneeberg A., Teasell R., Yao J., Eng J.J. (2021). Efficacy of an exoskeleton-based physical therapy program for non-ambulatory patients during subacute stroke rehabilitation: A randomized controlled trial. J. Neuroeng. Rehabil..

[135] Kim C., Kim H.-J. (2022). Effect of robot-assisted wearable exoskeleton on gait speed of post-stroke patients: A systematic review and meta-analysis of a randomized controlled trials. Phys. Ther. Rehabil. Sci..

[136] Nolan K.J., Karunakaran K.K., Roberts P., Tefertiller C., Walter A.M., Zhang J., Leslie D., Jayaraman A., Francisco G.E. (2021). Utilization of robotic exoskeleton for overground walking in acute and chronic stroke. Front. Neurorobot..

[137] Buesing C., Fisch G., O’Donnell M., Shahidi I., Thomas L., Mummidisetty C.K., Williams K.J., Takahashi H., Rymer W.Z., Jayaraman A. (2015). Effects of a wearable exoskeleton stride management assist system (sma^®^) on spatiotemporal gait characteristics in individuals after stroke: A randomized controlled trial. J. Neuroeng. Rehabil..

[138] Jacksteit R., Stöckel T., Behrens M., Feldhege F., Bergschmidt P., Bader R., Mittelmeier W., Skripitz R., Mau-Moeller A. (2021). Low-load unilateral and bilateral resistance training to restore lower limb function in the early rehabilitation after total knee arthroplasty: A randomized active-controlled clinical trial. Front. Med..

[139] Paterno M.V., Rauh M.J., Schmitt L.C., Ford K.R., Hewett T.E. (2012). Incidence of contralateral and ipsilateral anterior cruciate ligament (acl) injury after primary acl reconstruction and return to sport. Clin. J. Sport Med..

[140] Green L.A., Gabriel D.A. (2018). The effect of unilateral training on contralateral limb strength in young, older, and patient populations: A meta-analysis of cross education. Phys. Ther. Rev..

[141] Lim H., Madhavan S. (2023). Non-paretic leg movements can facilitate cortical drive to the paretic leg in individuals post stroke with severe motor impairment: Implications for motor priming. Eur. J. Neurosci..

[142] Peng W.W., Tang Z.Y., Zhang F.R., Li H., Kong Y.Z., Iannetti G.D., Hu L. (2019). Neurobiological mechanisms of tens-induced analgesia. Neuroimage.

[143] Vahdat S., Darainy M., Thiel A., Ostry D.J. (2019). A single session of robot-controlled proprioceptive training modulates functional connectivity of sensory motor networks and improves reaching accuracy in chronic stroke. Neurorehabil. Neural Repair.

[144] Ambreen H., Tariq H., Amjad I. (2020). Effects of bilateral arm training on upper extremity function in right and left hemispheric stroke. J. Pak. Med. Assoc..

[145] Stinear C.M., Byblow W.D., Steyvers M., Levin O., Swinnen S.P. (2006). Kinesthetic, but not visual, motor imagery modulates corticomotor excitability. Exp. Brain Res..

[146] Jarrassé N., Proietti T., Crocher V., Robertson J., Sahbani A., Morel G., Roby-Brami A. (2014). Robotic exoskeletons: A perspective for the rehabilitation of arm coordination in stroke patients. Front. Hum. Neurosci..

[147] Stoykov M.E., Corcos D.M., Madhavan S. (2017). Movement-based priming: Clinical applications and neural mechanisms. J. Mot. Behav..

[148] Lakens D., Stel M. (2011). If they move in sync, they must feel in sync: Movement synchrony leads to attributions of rapport and entitativity. Soc. Cogn..

[149] Aschersleben G. (2002). Temporal control of movements in sensorimotor synchronization. Brain Cogn..

[150] Bonassi G., Pellegrino L., Guggisberg A.G., Coscia M. (2023). Multimodal EEG and kinematic assessment of upper limb motor recovery early after stroke. Clin. EEG Neurosci..

[151] Sebastián-Romagosa M., Udina E., Ortner R., Dinarès-Ferran J., Cho W., Murovec N., Matencio-Peralba C., Sieghartsleitner S., Allison B.Z., Guger C. (2020). EEG biomarkers related with the functional state of stroke patients. Front. Neurosci..

[152] Chen C.-C., Lee S.-H., Wang W.-J., Lin Y.-C., Su M.-C. (2017). EEG-based motor network biomarkers for identifying target patients with stroke for upper limb rehabilitation and its construct validity. PLoS ONE.

[153] Noble J.W., Eng J.J., Boyd L.A. (2014). Bilateral motor tasks involve more brain regions and higher neural activation than unilateral tasks: An fmri study. Exp. Brain Res..

[154] Repp B.H., Su Y.-H. (2013). Sensorimotor synchronization: A review of recent research (2006–2012). Psychon. Bull. Rev..

[155] Mu Y., Huang Y., Ji C., Gu L., Wu X. (2018). Auditory over visual advantage of sensorimotor synchronization in 6- to 7-year-old children but not in 12- to 15-year-old children and adults. JExPH.

[156] O’Connell R.G., Kelly S.P. (2021). Neurophysiology of human perceptual decision-making. Annu. Rev. Neurosci..

[157] Wilken S., Böttcher A., Adelhöfer N., Raab M., Beste C., Hoffmann S. (2024). Neural oscillations guiding action during effects imagery. Behav. Brain Res..

[158] Guertin P.A. (2013). Central pattern generator for locomotion: Anatomical, physiological, and pathophysiological considerations. Front. Neurol..

[159] Shachykov A., Henaff P., Shachykov A., Popov A., Shulyak A. Neuro-musculoskeletal simulator of human rhythmic movements. Proceedings of the 2017 IEEE First Ukraine Conference on Electrical and Computer Engineering (UKRCON).

[160] Waller S.M., Whitall J. (2008). Bilateral arm training: Why and who benefits?. NeuroRehabilitation.

[161] Van Delden A., Peper C., Beek P., Kwakkel G. (2012). Unilateral versus bilateral upper limb exercise therapy after stroke: A systematic review. J. Rehabil. Med..

[162] Nakada K., Asai T., Amemiya Y. (2003). An analog cmos central pattern generator for interlimb coordination in quadruped locomotion. IEEE Trans. Neural Netw..

[163] Grillner S. (2006). Biological pattern generation: The cellular and computational logic of networks in motion. Neuron.

[164] MacKay-Lyons M. (2002). Central pattern generation of locomotion: A review of the evidence. Phys. Ther..

[165] Morquette P., Verdier D., Kadala A., Féthière J., Philippe A.G., Robitaille R., Kolta A. (2015). An astrocyte-dependent mechanism for neuronal rhythmogenesis. Nat. Neurosci..

[166] Williams C.A., DeWeerth S.P. (2007). A comparison of resonance tuning with positive versus negative sensory feedback. Biol. Cybern..

[167] Sasada S., Tazoe T., Nakajima T., Futatsubashi G., Ohtsuka H., Suzuki S., Zehr E.P., Komiyama T. (2016). A common neural element receiving rhythmic arm and leg activity as assessed by reflex modulation in arm muscles. J. Neurophysiol..

[168] Balter J.E., Zehr E.P. (2007). Neural coupling between the arms and legs during rhythmic locomotor-like cycling movement. J. Neurophysiol..

[169] Hosp J.A., Luft A.R. (2011). Cortical plasticity during motor learning and recovery after ischemic stroke. Neural Plast..

[170] McDonnell M.N., Koblar S., Ward N.S., Rothwell J.C., Hordacre B., Ridding M.C. (2015). An investigation of cortical neuroplasticity following stroke in adults: Is there evidence for a critical window for rehabilitation?. BMC Neurol..

[171] Sleimen-Malkoun R., Temprado J.-J., Thefenne L., Berton E. (2011). Bimanual training in stroke: How do coupling and symmetry-breaking matter?. BMC Neurol..

[172] Sawaki L., Boroojerdi B., Kaelin-Lang A., Burstein A.H., Bütefisch C.M., Kopylev L., Davis B., Cohen L.G. (2002). Cholinergic influences on use-dependent plasticity. J. Neurophysiol..

[173] Sawaki L., Wu C.W.-H., Kaelin-Lang A., Cohen L.G. (2006). Effects of somatosensory stimulation on use-dependent plasticity in chronic stroke. Stroke.

[174] Wang Y., Xie H., Sun H., Ren L., Jiang H., Chen M., Dong C. (2024). Influencing factors of psychological resilience in stroke patients: A systematic review and meta-analysis. Arch. Clin. Neuropsychol..

[175] Tan Z., Dong F., Wu L., Feng Y., Zhang M., Zhang F. (2023). Transcutaneous electrical nerve stimulation (tens) alleviates brain ischemic injury by regulating neuronal oxidative stress, pyroptosis, and mitophagy. Mediat. Inflamm..

[176] Mustin M., Hensel L., Fink G.R., Grefkes C., Tscherpel C. (2024). Individual contralesional recruitment in the context of structural reserve in early motor reorganization after stroke. NeuroImage.

[177] Grefkes C., Nowak D.A., Eickhoff S.B., Dafotakis M., Küst J., Karbe H., Fink G.R. (2008). Cortical connectivity after subcortical stroke assessed with functional magnetic resonance imaging. Ann. Neurol..

[178] Baker A., Schranz C., Seo N.J. (2023). Associating functional neural connectivity and specific aspects of sensorimotor control in chronic stroke. Sensors.

[179] Bellaiche S., Ibarolla D., Redouté J., Comte J.-C., Medée B., Arsenault L., Mayel A., Revol P., Delporte L., Cotton F. (2022). Upper limb rehabilitation after stroke: Constraint versus intensive training. A longitudinal case-control study correlating motor performance with fmri data. bioRxiv.

[180] Kim H., Park G., Shin J.-H., You J.H. (2020). Neuroplastic effects of end-effector robotic gait training for hemiparetic stroke: A randomised controlled trial. Sci. Rep..

[181] Caligiore D., Pezzulo G., Baldassarre G., Bostan A.C., Strick P.L., Doya K., Herreros I. (2017). Consensus Paper: Towards a Systems-Level View of Cerebellar Function: The Interplay Between Cerebellum, Basal Ganglia, and Cortex. Cerebellum.

[182] Ferrari P.F., Gerbella M., Coudé G., Rozzi S. (2018). Two Different Mirror Neuron Networks: The Sensorimotor (Hand) and Limbic (Face) Pathways. Neuroscience.

[183] Oldrati V., Schutter D.J. (2018). Targeting the human cerebellum with transcranial direct current stimulation to modulate behavior: A meta-analysis. Cerebellum.

[184] Suppa A., Quartarone A., Siebner H., Chen R., Di Lazzaro V., Del Giudice P., Ziemann U. (2017). The associative brain at work: Evidence from paired associative stimulation studies in humans. Clin. Neurophysiol..

[185] Castel-Lacanal E., Tarri M., Loubinoux I., Gasq D., de Boissezon X., Marque P., Simonetta-Moreau M. (2019). Transcranial magnetic stimulation in brain injury. Ann. Phys. Rehabil. Med..

[186] Tilp M., Ringler S., Mariacher H., Rafolt D. (2023). Unilateral strength training after total knee arthroplasty leads to similar or better effects on strength and flexibility than bilateral strength training—A randomized controlled pilot study. J. Rehabil. Med..

[187] Song J.S., Yamada Y., Kataoka R., Hammert W.B., Kang A., Spitz R.W., Wong V., Seffrin A., Kassiano W., Loenneke J.P. (2024). Does unilateral high-load resistance training influence strength change in the contralateral arm also undergoing high-load training?. Scand. J. Med. Sci. Sports.

[188] Coratella G., Galas A., Campa F., Pedrinolla A., Schena F., Venturelli M. (2022). The eccentric phase in unilateral resistance training enhances and preserves the contralateral knee extensors strength gains after detraining in women: A randomized controlled trial. Front. Physiol..

[189] Di Brino E., Ciavarro M., Iosa M., Sasso E., Prosperini L., Crispino V., Russo S., Morone G., Stampacchia G., Paolucci S. (2024). Preserved practice-dependent online motor learning and interlimb transfer in chronic stroke survivors: A novel quick and safe clinical assessment tool. Neurol. Sci..

[190] Merrick C.M., Doyle O.N., Gallegos N.E., Irwin Z.T., Olson J.W., Gonzalez C.L., Knight R.T., Ivry R.B., Walker H.C. (2024). Differential contribution of sensorimotor cortex and subthalamic nucleus to unimanual and bimanual hand movements. Cereb. Cortex.

