# Supplementary material for: Post-Stroke Rehabilitation: Neurophysiology Processes of Bilateral Movement Training and Interlimb Coupling—A Systematic Review
Source: J Clin Med. 2025 May 27;14(11):3757. doi: 10.3390/jcm14113757 (PMC12156210; doi:10.3390/jcm14113757)
Supplement: Supplementary file 1 [file jcm-14-03757-s001.zip › jcm-3530459-supplementary file S2.pdf]

Supplement II. Risk of BIAS assessment of the included research papers

STUDY	Total NOS Score	Type of study	**Risk of Bias Level**	**RoB 2.0 domain scores**/ Cochrane ROBINS-I
Bruyneel et al. (2021) [71]	6/9	Observational/ Experimental	**Moderate** (Good selection methods, moderate comparability due to limited confounder control, strong objective outcome measures, cross-sectional design limitations).	
Dhakate & Bhattad (2020) [72]	8/9	Observational	**Low** (Strong selection methods, moderate comparability due to limited control of confounders, rigorous objective outcome assessment, clear follow-up, minimal attrition).	
Duff et al. (2022) [73]	7/9	Observational	**Low-to-Moderate** (Strong participant selection, good objective outcome measurement, moderate comparability due to limited statistical control for confounders, cross-sectional design limiting follow-up assessment).	
Han & Kim (2016) [29]		Randomized Control Trial	**Some concerns**<br><br>This is primarily due to unclear randomization methods and a lack of explicit blinding of assessors, participants, and therapists. However, the study reports results with complete outcome data.	Domain 1: Some concerns<br>Domain 2: Some concerns<br>domain 3: Low risk<br>Domain 4: Some Concerns<br>Domain 5:<br>Low risk
Itkonen et al. (2019) [74]		Quasi-experimental (non-randomized) study	**Moderate/ High** Primarily due to unclear randomization and	Domain 1: High<br>Domain 2: Moderate<br>domain 3: Low risk<br>Domain 4: Moderate

			blinding methodology.	Domain 5: Low risk
Kim et al. (2022) [75]	7/9	observational, comparative design	**Moderate Quality** (7/9 NOS score), primarily impacted by uncertainties in selection and comparability.	
Kumagai et al. (2022) [76]		Randomized control trial	**Moderate Risk of Bias** Primarily due to issues related to blinding of therapists, participants, and assessors, common in rehabilitation interventions.	Domain 1: Low<br>Domain 2: Some concerns<br>domain 3: Low risk<br>Domain 4: Some concerns<br>Domain 5: Low risk
Lee et al. (2017) [38]		Randomized control trial	**Moderate Risk of Bias** Primarily due to unclear details of randomization and allocation concealment, as well as lack of explicit assessor blinding and inability to blind therapists and participants.	Domain 1: Some concerns<br>Domain 2: Some concerns<br>domain 3: Low risk<br>Domain 4: Some concerns<br>Domain 5: Low risk
Meng et al. (2018) [77]		Randomized controlled Trial	**Moderate Risk of Bias** Primarily attributed to potential deviations from intervention due to lack of blinding of participants and therapists, and unclear blinding of assessors.	Domain 1: Low<br>Domain 2: Some concerns<br>domain 3: Low risk<br>Domain 4: Some concerns<br>Domain 5: Low risk
Kaupp et al. (2018) [24]		Randomized controlled Trial	**Moderate Risk of Bias** Mainly due to the inability to blind participants and therapists, and the unclear blinding status of outcome assessors.	Domain 1: Low<br>Domain 2: Some concerns<br>domain 3: Low risk<br>Domain 4: Some concerns<br>Domain 5: Low risk
Lin et al. (2015) [78]		Randomized controlled Trial	**Moderate Risk of Bias** Primarily due to limitations in blinding procedures and unclear assessor blinding for outcome measurement.	Domain 1: Low<br>Domain 2: Some concerns<br>domain 3: Low risk<br>Domain 4: Some concerns<br>Domain 5: Low risk
Rodrigues et al. (2016) [79]		Randomized Controlled Trial	**Moderate Risk of Bias**	Domain 1: Low<br>Domain 2: Some

			Primarily related to lack of clarity regarding assessor blinding and inevitable challenges related to participant and therapist blinding.	concerns<br>domain 3: Low risk<br>Domain 4: Some concerns<br>Domain 5:<br>Low risk
Song, G.B. (2015) [80]		RCT	**Moderate Risk of Bias**<br>Primarily due to unclear details regarding randomization procedures and potential absence of blinding of outcome assessors, participants, and therapists.	Domain 1: Some concerns<br>Domain 2: Some concerns<br>domain 3: Low risk<br>Domain 4: Some concerns<br>Domain 5:<br>Low risk
Van Delden et al. (2015) [81]		Randomized controlled Trial	**Low Risk of Bias**<br>Only minor concerns due to inability to blind participants and therapists fully, which is common in rehabilitation interventions.	Domain 1: Low<br>Domain 2: Some concerns<br>domain 3: Low risk<br>Domain 4: Low<br>Domain 5:<br>Low risk
Abdollahi et al. (2018) [82]		Randomized controlled Trial	**Low Risk of Bias**<br>(Minor Concerns)<br>The only significant concern is performance bias arising from the unavoidable absence of blinding in therapy-based interventions.	Domain 1: Low<br>Domain 2: Some concerns<br>domain 3: Low risk<br>Domain 4: Low<br>Domain 5:<br>Low risk
Huang et al. (2022) [83]		Pilot experimental study (non-randomized or unclear randomization, single-session intervention)	**Moderate Risk of Bias**<br>Primarily due to unclear details regarding randomization and the inherent difficulty in blinding participants and assessors in training interventions.	
Li et al. (2023) [13]		Narrative/Not an RCT.Considered for Removal from the review	**Moderate to High Risk**<br>Primarily due to unclear literature search and selection processes, absence of explicit quality assessments, and limited transparency regarding potential biases.	Domain 1: Moderate<br>Domain 2: High Risk<br>domain 3: Moderate<br>Domain 4: High<br>Domain 5:<br>Moderate/High

Jayasinghe et al. (2021) [84]	6.5/9	Cross-sectional observational study	**Moderate Risk**<br>Mainly due to limited sample size and partial control of confounding factors (e.g., lesion characteristics and rehab history).	
Ardestani et al. (2020) [85]	8/9	Prospective cohort intervention study.	**Low Risk**	
Jo, P.Y. (2019) [86]	6/9	Dissertation/ Cross-sectional observational study	**Moderate:**<br>Limitations:<br>Cross-sectional design, no longitudinal follow-up.<br>Limited detailed control for potential confounding factors beyond basic demographics.	
Kwong et al. (2018) [87]		RCT	**Low Risk of Bias**	Domain 1: Low<br>Domain 2: Low Risk<br>domain 3: Low<br>Domain 4: Low<br>Domain 5:<br>Low
Arya et al. (2020) [15]		Pilot Randomized Controlled Trial (RCT)	**Low Risk of Bias**<br>This pilot RCT has high methodological rigor, indicating robust quality suitable for inclusion in systematic reviews and meta-analyses.	Domain 1: Low<br>Domain 2: Low Risk<br>domain 3: Low<br>Domain 4: Low<br>Domain 5:<br>Low
Klarner et al. (2016) [25]		Interventional (Pre-post design) Experimental Study	**Low to Moderate Risk of Bias**	Cochrane ROBINS-I<br>1.Bias/ Low<br>2.Bias. Moderate<br>3.Bias /Low<br>4.Bias/ Low<br>5.Bias./ Low<br>6.Bias./ Low<br>7.Bias./Low
Stoykov et al. (2020) [88]		Randomized Controlled Pilot Study (RCT)	**Low risk of Bias**	Domain 1: Low<br>Domain 2: Low Risk<br>domain 3: Low<br>Domain 4: Low<br>Domain 5:<br>Low
